# Deep learning can predict global earthquake-triggered landslides

**DOI:** 10.1093/nsr/nwaf179

**Published:** 2025-05-09

**Authors:** Xuanmei Fan, Xin Wang, Chengyong Fang, John D Jansen, Lanxin Dai, Hakan Tanyas, Nan Zang, Ran Tang, Qiang Xu, Runqiu Huang

**Affiliations:** State Key Laboratory of Geohazard Prevention and Geoenvironment Protection, Chengdu University of Technology, Chengdu 610059, China; State Key Laboratory of Geohazard Prevention and Geoenvironment Protection, Chengdu University of Technology, Chengdu 610059, China; State Key Laboratory of Geohazard Prevention and Geoenvironment Protection, Chengdu University of Technology, Chengdu 610059, China; GFÚ Institute of Geophysics, Czech Academy of Sciences, Prague 117 20, Czechia; State Key Laboratory of Geohazard Prevention and Geoenvironment Protection, Chengdu University of Technology, Chengdu 610059, China; Faculty of Geo-Information Science and Earth Observation (ITC), University of Twente, Enschede 7522 NB, Netherlands; Department of Earth and Space Sciences, Southern University of Science and Technology, Shenzhen 518055, China; School of Architecture and Civil Engineering, Chengdu University, Chengdu 610106, China; State Key Laboratory of Geohazard Prevention and Geoenvironment Protection, Chengdu University of Technology, Chengdu 610059, China; State Key Laboratory of Geohazard Prevention and Geoenvironment Protection, Chengdu University of Technology, Chengdu 610059, China

**Keywords:** earthquake-triggered landslide, global database, deep learning, landslide prediction model

## Abstract

Earthquake-triggered (coseismic) landsliding is among the most lethal of disasters, and rapid response is crucial to prevent cascading hazards that further threaten lives and infrastructure. Current prediction approaches are limited by oversimplified physical models, regionally focused databases, and retrospective statistical methods, which impede timely and accurate hazard assessments. To overcome these constraints, we developed the first comprehensive global database of ∼400 000 landslides associated with 38 of the most catastrophic earthquakes over the past 50 years. Leveraging this extensive dataset, we developed advanced deep-learning models that predict the probability of landsliding for any earthquake worldwide with an average spatial accuracy of ∼82% in less than a minute, without relying on prior local knowledge. Our framework enables swift disaster evaluation during the critical early hours following an earthquake while also enhancing pre-event hazard planning. This study offers a scalable and efficient tool to mitigate the catastrophic impacts of earthquake-triggered landslides, representing a transformative advance in global geohazard prediction.

## INTRODUCTION

Earthquakes are among the most persistent natural catastrophes, causing severe loss of life and abrupt economic disruption [1]. Major earthquakes (magnitude, *M*_w_ ≥ 7) occur on average every month worldwide (https://www.usgs.gov/programs/earthquake-hazards/lists-maps-and-statistics) and have claimed ∼750 000 lives in the two decades 1998–2017 (https://www.who.int/health-topics/earthquakes#tab=tab_1). Earthquake-triggered (coseismic) landslides (abbreviated to ‘landslides’ hereafter) are the third largest cause of associated fatalities—after building collapse and tsunami flooding [2,3]—and specifically in mountainous landscapes they intensify hazardous congruences of earthquakes [4]. For example, the *M*_w_ 7.9 Wenchuan (China) earthquake triggered nearly 200 000 landslides [5], killing >20 000 people (>30% of the death-toll), and causing USD 30 billion in economic losses (25% of the total) [6]. The *M*_w_ 7.9 Gorkha (Nepal) earthquake induced >25 000 landslides and debris flows, which caused more than 40% of the total 9000 fatalities [7]. Landslides also frequently delay rescue responses to earthquake-affected regions, greatly amplifying loss of life and suffering [8].

While rapid response is fundamental for limiting casualties and mitigating the multi-hazard chains that stem from catastrophic earthquakes [9], the first priority in mountain regions is to evaluate: where are the landslides? Remote sensing is a notable recent advance over field reconnaissance [10,11], but even so, the timely availability of cloud-free, large-scale imagery depends on favorable weather and satellite trajectories [12]. In answer to such urgent challenges, a real-time predictive model of potential landslide distribution would greatly assist preliminary assessments of hazards immediately post-earthquake prior to the arrival of remote-sensing data (sometimes delayed by several days or even weeks) [13]. Such a tool would bolster appreciably the resilience of mountain populations to earthquake hazards [14].

Research efforts aimed at predicting landslides can be broadly classified into physics-based and data-driven models [15]. Physics-based models analyze hillslope deformation and rupture under the force of earthquakes via mechanical analysis. An early key example, the Newmark method [16], was devised at the laboratory scale to assess coseismic ruptures on hillslopes and the likelihood of landslides. Jibson, Harp and Michael [17] built upon this approach to accommodate failure at landslide-scale for the first time. While these physics-based approaches allow for intuitive observation and simulation of the deformation characteristics and propagation of landslides, their practical application for analyzing extensive landslides simultaneously at large scale is constrained by their detailed physical parameter requirements, which demand comprehensive understanding of the physical mechanisms [18]. Data-driven models, by contrast, aim to reveal the distribution pattern of landslides via statistical analysis of past earthquake events. This approach predicts potential landslides under differing seismic conditions by establishing a functional relationship between the probability of landslide occurrence and known or inferred instability factors [19,20]. Compared to the physics-based approach, data acquisition and processing demands are notably less for data-driven models. Consequently, a large number of data-driven models have been proposed by learning from the training samples derived from the growing catalogue of past events [21,22].

Forecasting landslides involves determining the likelihood of ‘when’ they will occur and ‘where’ they will occur (the latter is also known as susceptibility). Accurately and promptly predicting landslides is challenging due to event-to-event variability and model applicability [23]. Current limitations with landslide prediction can be framed in terms of three general perspectives: (i) traditional statistical analyses [24] and machine-learning algorithms [25] both typically establish a linear (or non-linear) mapping relationship between landslide likelihood and selected indicators, which often leads to overprediction of the spatial footprint of landslide-affected hillslopes [26]. Deep learning, by contrast, is capable of abstracting higher-level features from input factors without intervention, providing superior fitting capabilities with a complex model structure [27,28]. (ii) Susceptibility analysis, as conventionally applied, is based on models trained in hindsight, which is not strictly prediction. If the goal is to hasten emergency rescue globally, the required model is one that can predict landslides without any prior local labels. (iii) Most control indicators adopted as model inputs are prone to being event specific, as opposed to universal. Therefore, identifying control indicators that perform well at regional- or even global-scale would be a major advance. With this in mind, we should begin first of all with the question of whether it is feasible for a regional- or even global-scale landslide predictive model to be derived.

With the aim of investigating how and why landslides develop during earthquakes under differing boundary conditions, we compiled a representative global selection of nearly 400 000 coseismic landslides from the past, establishing the largest catalog to date. Fourteen primary control indicators (PCIs) governing large-scale landsliding were identified and a deep learning-based algorithm was devised to test the validity of a regional- versus global-scale model for predicting landslides triggered by earthquakes. We demonstrate that our predictive model is indeed feasible and performs well at the global scale, heralding a much-anticipated advance in the geohazard sciences.

## RESULTS

### Model construction of global earthquake-triggered landslide prediction

**Figure 1.**
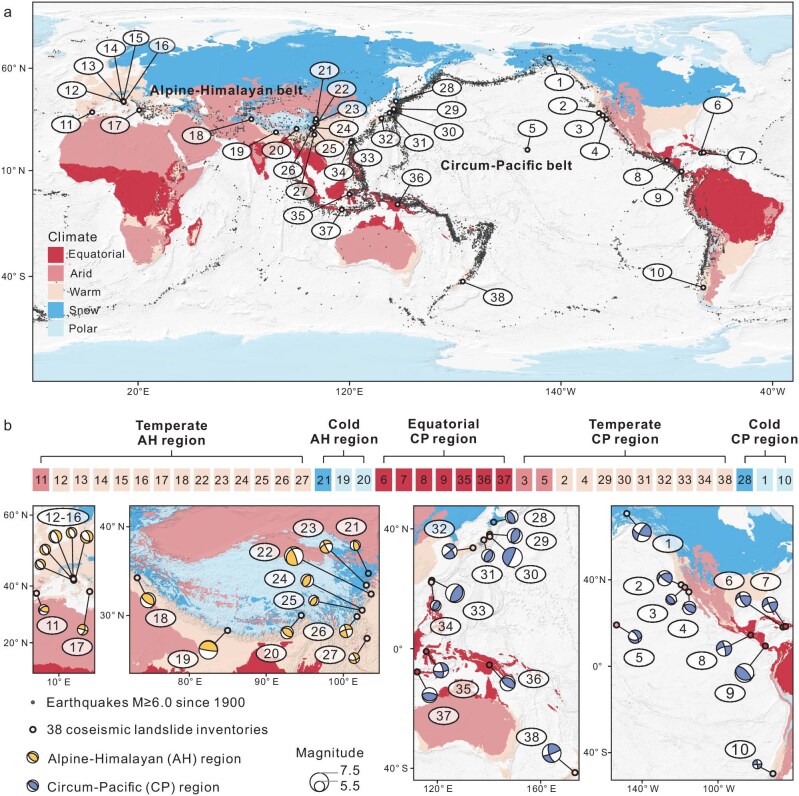
Worldwide distribution of earthquakes. (a) Coseismic landslide inventories within the Alpine-Himalayan and Circum-Pacific belts. (b) The enlarged view of highlighted 38 earthquakes. The five Köppen-Geiger climate zones are here simplified into three for regional division: cold (polar plus snow), temperate (warm plus arid), and equatorial. Beachballs signify primary seismic rupture dynamics.

The most intensive coseismic landsliding worldwide falls broadly along two primary belts marking collisional plate tectonic boundaries: the Circum-Pacific (CP) belt and the Alpine-Himalayan (AH) belt [29]. We subdivide these two tectonic regions into three climatic zones [30]: cold, temperate and equatorial (Fig. 1).

From a global database of *M*_w_ ≥ 6 earthquakes compiled from previous studies [31], we selected 38 events (Fig. 1) with landslide inventories conforming to strict criteria for data quality (see Methods). The 38 earthquakes yielded a dataset of 398 698 landslides mapped within the CP and AH belts (Fig. 1; see Table S1). From this dataset we identified (see Methods and Supplementary Note 1) 14 PCIs held to govern landslide susceptibility based on: topography (slope, aspect, relief, terrain roughness, plan curvature, topographic position index [TPI]); geo-ecology (lithology, soil type, land cover, normalized difference vegetation index [NDVI]); hydrology (distance to river, topographic wetness index [TWI]); and seismology (distance to fault, peak ground acceleration [PGA]).

To optimize deep learning-based prediction, we implemented a three-step design. (i) Model training: an individual seismic event was isolated for testing with the remainder serving to train the models, ensuring prediction independence. (ii) Network architecture: we developed a multi-scale fully convolutional regression network (see Methods), which expands the spatial scope of feature extraction, and is beneficial for capturing both local and global triggering and environmental information. And (iii) model validation: we employed metrics based on a confusion matrix and receiver operating characteristic (ROC) from predictions versus ground truth comparison, including area under the ROC curve (AUC), F1 score, precision, recall, overall accuracy (ACC), and Kappa, to evaluate model performance.

In order to demonstrate the predictive capabilities of our model on regional and global scales, we selected a representative earthquake from each of the five regions (Fig. 1) as a case study using the two models. From the CP belt, we selected the 2021 Nippes earthquake (equatorial, Haiti), the 2016 Kaikoura earthquake (temperate, Aotearoa New Zealand), and the 2002 Denali earthquake (cold, USA). From the AH belt we selected the 2022 Luding earthquake (temperate, China), and the 2015 Gorkha earthquake (cold, Nepal).

### How well do primary control indicators predict landslides?

**Figure 2.**
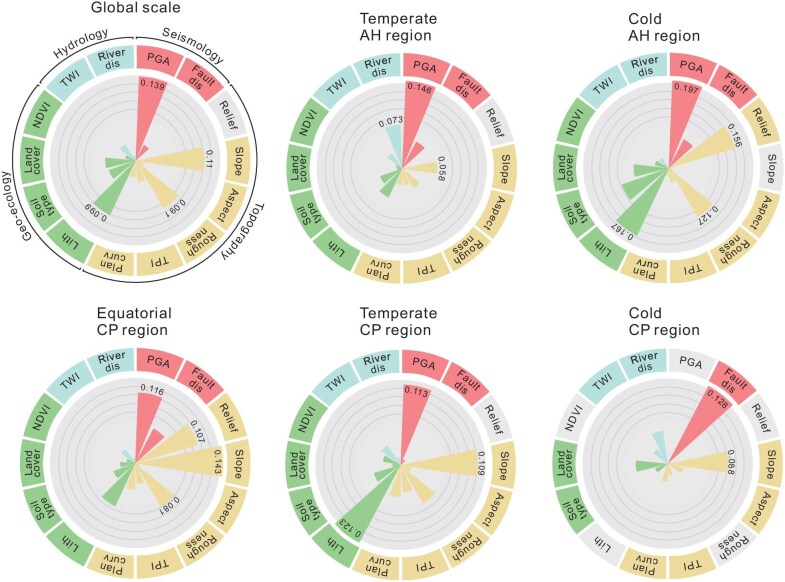
The variation of primary control indicator rankings for the global-scale model and five regional models within the Alpine-Himalayan and Circum-Pacific belts. Abbreviations: fault dis, distance to fault; river dis, distance to river; plan curv, plan curvature; lith, lithology.

The top three performing control indicators are PGA, hillslope angle, and lithology (Fig. 2) in each region; these three remain at the crux of whether landsliding will occur, or not, as they directly translate the sudden triggering force (PGA), the gravity force (hillslope angle), and the ruptured material (lithology). Relief (strongly correlated with slope) and terrain roughness also perform strongly in most regions. Six indicators (soil type, distance to fault, TPI, land cover, TWI, and plan curvature) are moderate performers, and three indicators (distance to river, NDVI, and aspect) are weakest (Fig. 2).

### Deep learning applied to landslide prediction

**Figure 3.**
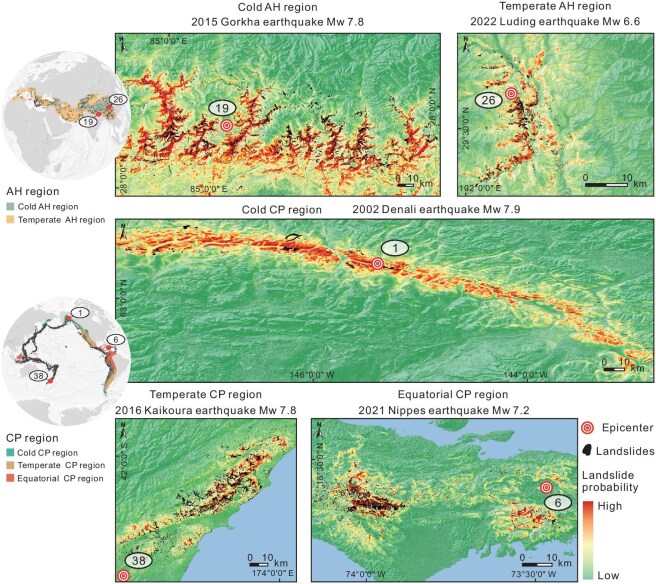
Landslide predictions of the optimal models for five major earthquake events.

The predictive maps align closely with the actual landslides that occurred during the test seismic events (Fig. 3 for the optimal model and Fig. S1 for the underperformed model). Mean AUC and ACC of the global-scale predictions in these five cases reached 83.4% and 77.3%, which are remarkably high for independent scenarios devoid of any prior labels. Their average F1 and Kappa attained 77.5% and 0.546, respectively, thereby indicating a well-balanced performance for both high- and low-susceptibility areas. The overall performance of the regional-scale model is slightly better than that of the global scale, with average AUC, ACC, F1, and Kappa of 84.0%, 77.5%, 77.6%, and 0.551, respectively. The model based on global data contains a more comprehensive and varied database, yielding more balanced parameter training and ensuring a lower limit of prediction for any outcome. While the regional-scale model can optimize parameter weighting under similar background data and enhance the upper limit of targeted prediction, it is naturally more prone to the limiting effects of small sample size (see Table S2). This effect also accounts for the absence of some strong performers (e.g. PGA and lithology) among the PCIs in the cold CP model (Fig. 2). Supplementary Note 3 demonstrates the detailed performance of the global and regional models for each test event.

### Uncertainty arising from uneven data quality

Although the predictive performance is robust to the uncertainty of input data (e.g. landslide inventory and PGA) (see Supplementary Note 4), it is severely limited by the varying sources of inputs available. Most of our PCIs are derived from digital elevation models (DEMs), seismic parameters, and fault inventories—all of which are susceptible to the absence of satellite data, the deployment of seismological stations, and the accuracy of geological surveys, respectively. To illustrate our point, we take the 2022 Luding earthquake and draw three comparisons based on differing sources of regional-scale PCIs: an accurate data source versus a less accurate one, as follows: (i) DEMs generated from AW3D30 versus SRTM 90 m; (ii) PGA data from the China Earthquake Networks Center (CENC) versus the United States Geological Survey (USGS), and (iii) fault inventories from the China Active Faults Database (CAFD) versus the GEM Global Active Faults Database (see Supplementary Note 1).

**Figure 4.**
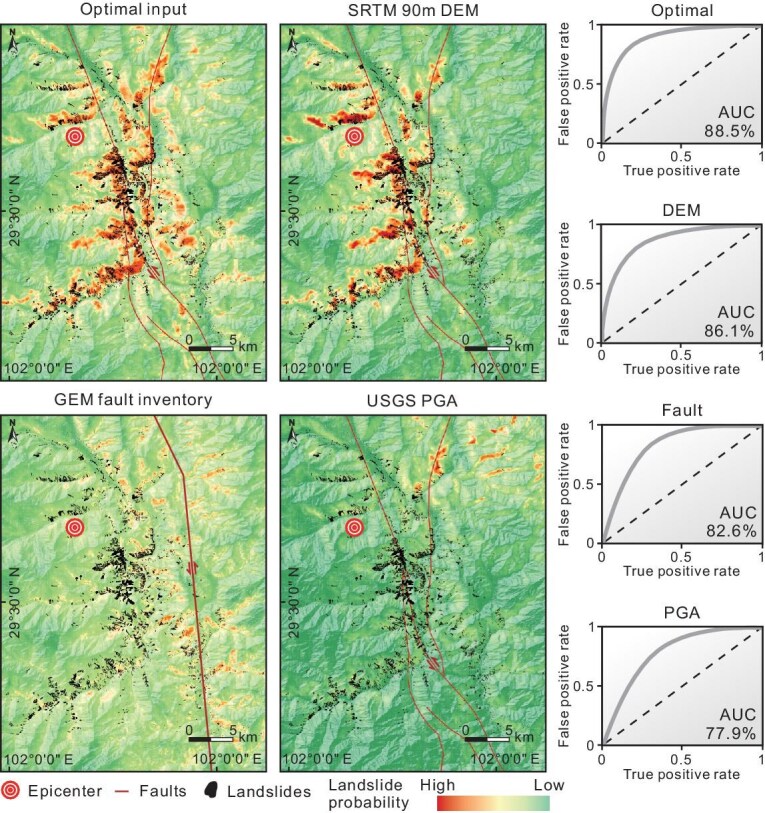
Predicted performances of the 2022 Luding event under various data qualities.

As expected, we find that the most favorable results are obtained when precise PCIs are utilized (AUC ∼88.5%). Based on our predictive landslide maps (Fig. 4), we find that: (i) susceptibility maps generated via DEM with lower-resolution (∼90 m) differ little from that generated via higher-resolution input (∼30 m). Certain susceptibility characteristics of a landslide may either become amplified to an entire pixel or get overwhelmed by the surroundings, thereby leading to a greater homogeneity of the predicted probabilities from one location to another. (ii) USGS-derived PGA causes an overall shift of the predicted landslide extents towards the northeast relative to those based on CENC data, thereby strongly limiting prediction performance (and reflecting the dominance of PGA among the PCIs). And (iii) GEM yields inaccurate spatial displacement of the mapped faults demonstrated by the CAFD. The less accurate data (90 m DEM, USGS PGA, and GEM fault inventory) produce a decrease in model AUC accuracy by 2.4%, 10.7%, and 14.1%, respectively. These findings underscore the significant influence of data quality on model performance, particularly when predicting landslide occurrence worldwide, where data sources exhibit considerable variability. This highlights the need for continuously updated, high-quality, and consistent datasets, while also emphasizing the necessity of incorporating uncertainty into predictive frameworks.

## DISCUSSION

### Overall performance of the global- and regional-scale model

The regional-scale models perform well in most scenarios, as is expected given the fact that they mirror local characteristics of landslide development under comparable environmental conditions. Their major limitation, however, is that they contain fewer event samples, which can lead to inadequate training and the potential for overfitting models in favor of events with more landslides (a notable issue with the two cold regions, CP and AH). By contrast, the global-scale model considers the development of landslides in different environments with abundant training samples. Accordingly, we settled upon a strategy that adopts regional-scale models where sufficient training events are available (i.e. in equatorial CP, AH, and temperate AH regions), while applying global-scale models in the two regions that lack sufficient training (i.e. cold CP and AH regions). We anticipate that the occurrence of major earthquakes in these two cold regions will provide new training samples that, over time, will strengthen the performance of the regional models.

**Figure 5.**
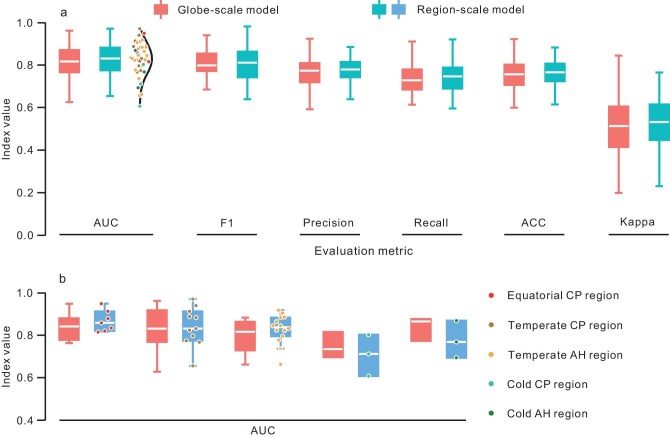
Overall performance of landslide prediction models. (a) Comprehensive accuracies of global and regional models. (b) AUC comparison between global and regional models within different regions.

To evaluate model performance considering all events, we adopted the leave-one-out cross-validation approach [32]. First, one landslide inventory is excluded as a test case, leaving the other inventories for model training; and second, the model is then applied to the landslide inventory that was excluded in the first step. Following an inversion approach, we ran the prediction model 38 times (once for each of our landslide inventories) at the global- and regional-scale, respectively, and then quantified model performance via the indices of AUC, precision, recall, F1, accuracy, and Kappa (Fig. 5; see Table S3).

Taking all historical events into account, the regional-scale model performs slightly better (1.15 ± 0.30%, 0.50 ± 0.49%, 0.80 ± 0.46%, and 1.61 ± 0.92% higher for AUC, F1, ACC, and Kappa, respectively) than the global-scale model. However, from the perspective of individual regions the comparison shifts. We find that a regional-scale model performs better when applied to the equatorial CP, temperate CP, and temperate AH regions; hence, these models should be used for predicting future landslides. The global model, however, is the better choice for modelling events in the cold CP and AH regions.

### Advantages of a deep learning-based approach over machine learning

**Figure 6.**
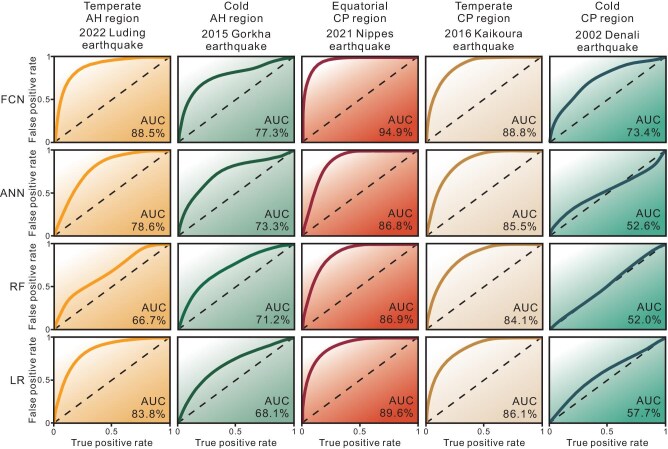
Predicted performances of five major earthquake events by using different modeling algorithms.

To further test our approach, we compared it with machine-learning algorithms applied in prior studies [25]; namely, Logistic Regression (LR), Random Forest (RF), and Artificial Neural Network (ANN)—while maintaining the same training setups. We find our deep learning approach outperforms the machine-learning algorithms, with notable AUC improvements of 5.4%–8.2%, 2.7%–4.7%, 15.7%–21.4%, 4.8%–21.8%, and 3.9%–9.1% for the 2021 Nippes event, 2016 Kaikoura event, 2002 Denali event, 2022 Luding event, and 2015 Gorkha event, respectively (Fig. 6).

The advantages of the deep learning-based approach for landslide prediction stems from its ability to automatically explore intricate coupling mechanisms and impacts based on inputs with physical implications, and to generate novel discriminative features for learning—albeit often being non-interpretable [33]. This mechanistic advantage distinguishes it from previous machine learning-based algorithms and underscores its exceptional success in landslide prediction. Our deep learning-based prediction model is capable of comprehensively investigating and comprehending the intricate correlation between triggering factors and environmental conditions, which has been a persistent challenge [34,35]. Accordingly, our approach is proficient in accurately predicting the landslide distribution of earthquakes worldwide.

### Our model tested with a hypothetical *M*_w_ 7.5 earthquake scenario

To underscore the critical role of coseismic landslide prediction and the advanced state of our deep learning-driven model, we simulated a scenario of the devastating *M*_w_ 7.5 earthquake. Our simulated scenario (see Supplementary Note 5) targets the Anninghe fault in Mianning, China (epicenter: 102.30°E, 28.54°N), a densely populated mountainous area located within the temperate AH belt. Employing the advanced prediction capabilities of our regional model, the spatial probability of earthquake-triggered landslides was determined in just 34 seconds—potentially several days ahead of the availability of large-scale imagery. When this real-time predictive data is overlaid with population and infrastructure within the seismic zone, the associated landsliding is shown to directly threaten ∼70 000 people (see Fig. S2) [36]. Prompt prediction of landslides serves as an invaluable tool for emergency management, offering critical guidance for hazard mitigation and potentially saving countless lives in the vital early hours of rescue efforts.

Despite the substantial advancements made by our models in predicting earthquake-triggered landslides, several challenges remain that must be addressed for broader applicability and deeper insight. On one hand, the non-interpretable nature of the deep learning-based model not only hinders understanding of the underlying physical mechanisms but also impedes generalizability to diverse and underrepresented regions. Future work should focus on developing a framework that couples physical mechanisms with data-driven learning, facilitating rapid and accurate predictions even under conditions of limited data and computational resources. This would enable the development of a robust, globally applicable model capable of delivering precise predictions in diverse environments. On the other hand, although our study focuses on coseismic landslides, the underlying multi-channel fully convolutional architecture can be readily repurposed for rainfall-induced events. By substituting seismic indicators (e.g. PGA and fault proximity) with hydrometeorological variables, such as cumulative antecedent rainfall, intensity-duration metrics, and soil moisture indices, the network enables one to learn the spatial thresholds governing rainfall-induced slope failure. Further, embedding sequential rainfall maps as a temporal channel would allow the model to capture the evolution of subsurface saturation and pore-pressure buildup. Together, these adaptations would yield rapid, high-resolution predictive maps for both seismic- and rainfall-induced hazards, offering a unified, operational tool that supports real-time emergency response and pre-event planning on a global scale.

## METHODS

### Global dataset of earthquake-triggered landslides

Abundant and diverse landslide training samples are the foundation of generalized prediction through data-driven methods. Herein, we conducted an extensive investigation of earthquakes exceeding *M*_w_ ≥ 6 worldwide since the 1970s. To optimize the utilization of current inventories while mitigating the influence of substandard data, we implemented three assessment criteria [23,33: (i) landslides were depicted as polygons rather than points; (ii) each polygon was covered by a landslide footprint entirely; and (iii) the boundary of each landslide was explicitly indicated. Consequently, we scrutinized 38 inventories possessing competent landslide records and rectified conspicuous missing and false identifications by incorporating remote sensing-based visual interpretation and sophisticated landslide intelligent recognition, which employs a change detection-based multiple classifier ensemble strategy [37], thereby ensuring that the labeled landslides followed rather than preceded the earthquake in question. Ultimately, we amalgamated the authenticated labels of the events, yielding a global database of 398 698 coseismic landslides, categorized into five classes (i.e. Equatorial CP, Temperate CP, Cold CP, Temperate AH, and Cold AH regions) with similar environmental backgrounds (see Table S1).

We identified a suite of 17 PCIs governing landslide susceptibility [38] based on topography (slope, aspect, relief, terrain roughness, plan curvature, profile curvature, TPI), geo-ecology (lithology, soil type, land cover, NDVI), hydrology (distance to river, TWI), and seismology (distance to fault, focal mechanism, PGA, peak ground velocity [PGV]) factors, where the extents were determined in accordance with the seismic-affected areas based on ShakeMaps [39]. The data configurations of landslide geospatial labels (comprising both landslide and non-landslide records) along with their corresponding attributes were transformed into raster maps with a spatial resolution of 30 m WGS 1984 geographic coordinate system in compliance with the initial topographical parameters. The utilized datasets are compiled in Table S4.

Based on the analysis of various events worldwide, landslide distributions exhibit distinct patterns under the 17 PCIs considered (see Fig. S3). The frequency of landslides correlates negatively with distances to faults and rivers. This reflects the contributions of strong seismic forces to slope collapse and demonstrates the decrease in shear strength of rock caused by fluvial incision, which influences long-term slope adjustment and stability. For most factors, the landslide frequencies present skewed distributions with concentration in specific intervals. For instance, landslides conditioned by slope and relief follow a typical (and similar) pattern, with frequency modes ranging 18–48° in slope and 400–1200 m in relief. Landslides follow similar patterns regarding PGA (modes spanning 0.20–0.45 g) and PGV (modes spanning 40–90 cm/s), which indicate the triggering aggregation of ground motion. Other factors (e.g. lithology, soil type, land cover, and NDVI) exhibit apparent correlations with landslide distribution within an earthquake-affected region. However, their natural attributes may vary across different seismic zones. The statistics of landslide frequencies with respect to most factors provide limited insights from a global perspective. Establishing an effective prediction approach requires quantitatively revealing the controlling factors for the landslides within a specific area of consideration.

### Primary control indicators of earthquake-triggered landslides

On one hand, a unified physical initiation mechanism suggests the plausibility of a universal landslide model [40]; but on the other hand, the interdependence of landslide-inducing factors varies from region to region, resulting in substantial disparities in the distribution, morphology, and scale of landslide development [41]. Considering this, an alternative approach is to develop models in diverse environments with consideration of the tectonic and climatic conditions. We targeted the two primary seismic belts; namely, the CP and AH belts, as a basis for model categorization in terms of tectonic environment [42]. These tectonic subdivisions were subsequently subdivided based on a global climate classification [30]. In accordance with the evaluated conditions, the collected landslide events were categorized into distinct regions for the purpose of region-specific model development.

Prior to establishing the global- and region-based models, it is imperative to circumvent multicollinearity, where multiple independent variables are linearly interrelated, resulting in flawed modeling and attenuated predictive capacity due to erroneous system analysis [43]. Consequently, to identify the optimal set of PCIs to use as inputs for model training, a multicollinearity analysis was carried out to assess the appropriateness of the inputs based on their non-independence. We employed an index, namely the variance inflation factor (VIF), which quantifies the extent to which collinearity increases the variance of an estimated regression coefficient. The degree of correlation between one variable and other variables in linear regression can be gauged via the *R*-squared (*R*^2^) value, where the variable of interest is predicted by the remaining factors [44]. The VIF for a variable can therefore be calculated as in Equation 1:


(1) \begin{eqnarray*}
\mathrm{VIF} = \frac{1}{{1 - \mathit{R}_{\it i}^2}}( {\it {i} = 1,2,\ldots,m}),
\end{eqnarray*}

where $R_i^2$ represents the coefficient between the *i*th independent variable and the other $m - 1$ independent variables. The greater the VIF value, the more pronounced the collinearity between a variable and the residual variables. Some studies prefer to use the tolerance (TOL) instead as in Equation 2, which is the reciprocal of VIF [45]:


(2) \begin{eqnarray*}
\mathrm{TOL} = 1 - \mathit{R}_{\it i}^2 = \frac{1}{\mathrm{VIF}}\left( {\it {i} = 1,2,\ldots,m} \right).
\end{eqnarray*}

A VIF value exceeding 10 or a TOL value below 0.1 indicates a possible multicollinearity scenario, where the variable is strongly correlated with the other variables, and the variable with the highest VIF or the lowest TOL value is eliminated [25]. We re-evaluated the VIF for the residual variables until they all satisfied the criteria. Considering the potential variations in landslide developmental approaches and predictive models, PCIs were selected separately for global and regional units.

To examine the outcomes of the multicollinearity analysis, Pearson's correlation coefficient *r*, a measurement of the linear correlation between any two independent variables, was calculated for the residual factors as in Equation 3 [46]:


(3) \begin{eqnarray*}
r = \frac{1}{{n - 1}}\mathop \sum \limits_{j = 1}^n \left( {\frac{{{X}_j}}{{{\sigma }_x}}} \right)\left( {\frac{{{Y}_j - \bar{Y}}}{{{\sigma }_y}}} \right),
\end{eqnarray*}

where *n* is sample size, ${X}_j$ and ${Y}_j$ are the individual samples of variable *X* and *Y* indexed with *j*, $\bar{X}$ and $\bar{Y}$ are the means of the samples of variable *X* and *Y*, while ${\sigma }_x$ and ${\sigma }_y$ are the standard deviations of the samples of variable *X* and *Y*. A linear correlation between two variables is deemed significant if the absolute value of their coefficient exceeds 0.6 [47]. In case of independent variables exhibiting high correlation, the one with a higher VIF value (as determined by multicollinearity analysis in the previous step) was eliminated until the desired standard was satisfied.

Subsequently, we evaluated the relative importance of the PCIs across various regions through the employment of information gain (IG), a rapid attribute ranking technique that serves to preliminarily demonstrate the explanatory potential of independent variables (i.e. PCIs) for the dependent variable (i.e. landslide). IG is a feature evaluation method that measures the anticipated reduction in entropy, which is a measurement of disorder or impurity, resulting from the partitioning of a dataset based on a given attribute [48]. Therefore, the IG value for a PCI ${P}_k$ and the landslide occurrence *Q* can be computed using Equation 4:


(4) \begin{eqnarray*}
IG\left( {Q,{P}_k} \right) = H\left( Q \right) - H\left( {Q{\mathrm{|}}{P}_k} \right),
\end{eqnarray*}

where $H( Q )$ denotes the entropy value of *Q*, and $H(Q|{P}_k)$ denotes the entropy of *Q* conditioned on a PCI ${P}_k$. Hence, the PCIs providing more information have higher IG values.

### Earthquake-triggered landslide prediction model

The dataset spans ∼50 years during which remote sensing technology has advanced. Accordingly, all landslide labels as well as PCI inputs were resampled to a uniform 30 m resolution, ensuring data consistency and enabling subsequent predictive modeling. To address any potential bias arising from the overrepresentation of landslides from a single event, we applied a refined labeling approach. In areas identified as landslides, the label was set to 1, while in background regions values fall between 1 and 0, as defined by the Poisson equation with Dirichlet boundary conditions (see Supplementary Note 2). The input labels were generated through a sliding window approach with 20% spatial overlap across seismic-affected regions. Each window consists of 448 × 448 × *m* pixels, corresponding to 13.44 km × 13.44 km at 30 m resolution, with *m* denoting the number of input channels. These patches along with their associated PCIs were then used as training samples. For the region-based model, we selected an individual event for testing purposes, with the remaining events serving as the training set, thereby ensuring prediction independence. Similarly, in the construction of the global model, the test event of each region was combined to form a new global test set, while the samples of the remaining events were integrated as the new global training set. This strategy maintains the independence of event prediction while facilitating a comparison of results from both models.

To achieve accurate prediction of landslide events, we devised a multi-scale architecture via a fully convolutional regression network (see Fig. S4). Our model framework is meticulously designed to capture the fundamental conditions underpinning landslide occurrence. The input stratums are tailored to assimilate landslide attributes, where the input indicators are formatted as height *H*, width *W*, and feature count, and the feature dimension of the first layer is set as $N = 32$. The encoder component of the network is an amalgamation of a sequence of convolutional strata and pooling tiers, yielding a cascade of feature extraction cycles that engender a spatial matrix characterized by dimensions $H/4$, $W/4$, $16N$. To further refine the predicted efficacy, a convolutional block attention module (CBAM) is interwoven between the decoder and encoder segments [49]. This addition is instrumental in focusing on key information. In the decoder domain, a series of deconvolution layers and up-sampling layers are arranged, connecting with the encoded features for gradual decoding and reconstruction, ultimately producing an output layer result (*H, W*, 1).

To quantitatively assess the accuracy of model output, we utilized indicators based on confusion matrix and ROC, which is a two-dimensional plot of true positive rate on the Y-axis versus false positive rate on the X-axis. These indicators include the AUC, F1, precision, and recall, which serve to evaluate the performance of the predicted landslide target. AUC is a quantitative measure of model accuracy with a value between 0.5 and 1, where higher values indicate better performance. Furthermore, we computed the indices of ACC and Kappa coefficient, which take into account the non-landslide performance as well. It is worth noting that to prevent excessive non-landslide pixels from being included as validation data in a test event (which could lead to inflated indices), we considered those pixels covered entirely by landslides and an equal number of randomly selected non-landslide pixels with PGA exceeding 0.12 g for verification [50].

## Supplementary Material

nwaf179_Supplemental_File

## Data Availability

All data used in this study are included in this published article and its supplementary materials. The earthquake-triggered landslide predictions for the hypothetical and 38 historical cases are available on the website of the State Key Laboratory of Geohazard Prevention and Geoenvironment Protection (https://eqtl-prediction.cdut.edu.cn/dtglobe).

## CODE AVAILABILITY

The script of the earthquake-triggered landslide prediction approach is deposited at Zenodo (https://doi.org/10.5281/zenodo.11255154).
