# Supplementary material for: Deep learning can predict global earthquake-triggered landslides
Source: Natl Sci Rev. 2025 May 9;12(7):nwaf179. doi: 10.1093/nsr/nwaf179 (PMC12163991; doi:10.1093/nsr/nwaf179)
Supplement: nwaf179_Supplemental_File [file nwaf179_supplemental_file.docx]

*Supplementary Materials for*

**Deep learning can predict global earthquake-triggered landslides**

Xuanmei Fan^1,†,*^, Xin Wang^1,†^, Chengyong Fang^1,†^, John D. Jansen^2^, Lanxin Dai^1^, Hakan Tanyas^3^, Nan Zang^4^, Ran Tang^5^, Qiang Xu^1^, Runqiu Huang^1^

^1^ State Key Laboratory of Geohazard Prevention and Geoenvironment Protection, Chengdu University of Technology; Chengdu, China.
^2^ GFÚ Institute of Geophysics, Czech Academy of Sciences; Prague, Czechia.
^3^ Faculty of Geo-Information Science and Earth Observation (ITC), University of Twente; Enschede, Netherlands.
^4^ Department of Earth and Space Sciences, Southern University of Science and Technology; Shenzhen, China.
^5^ School of Architecture and Civil Engineering, Chengdu University; Chengdu, China.

^*^ Corresponding author: Xuanmei Fan (fxm_cdut@qq.com)

^†^ Equally contributed to this work.

**This PDF file includes:**

Supplementary Notes 1 to 5

Supplementary Figs 1 to 6

Supplementary Tables 1 to 5

References

Supplementary Note 1. Primary control indicators of earthquake-triggered landslides

From the perspective of landslide triggering mechanisms, we considered 17 factors broadly acknowledged in previous studies. Topography-related factors—namely, slope, aspect, relief, terrain roughness, plan curvature, profile curvature, and TPI—were extracted based on ALOS AW3D30 DEM^1^ using TopoToolbox, a widely used open-source program for topographic analysis and modeling^2^. For geo-ecological parameters, lithology and soil type were directly obtained from Global Lithology Map (GLiM)^3^ and FAO Soil Map^4^, respectively. Land cover was obtained from the GLC_FCS30D^5^ by using, for each earthquake, the pre-event product that was most temporally proximate. NDVI was calculated from the most recent pre-earthquake multispectral imagery acquired by Landsat 4–8 satellites. Due to the fact that many smaller rivers are often omitted in global river datasets, hydrological parameters such as distance to river and TWI were derived by first extracting river network information with DEM and TopoToolbox, and then creating buffered zones and applying calculations to determine the corresponding distances and TWI values, respectively. Regarding seismic parameters including focal mechanism, PGA, and PGV, we were able to obtain the necessary data post-earthquake, thanks to the extensive global monitoring and modeling efforts of the USGS ShakeMaps. However, in China, where USGS equipment is scarce, we instead utilized seismic monitoring and inversion parameters from the CENC. Similarly, fault data for China were sourced from the CAFD^6^, while fault data for other regions worldwide were obtained from the GEM^7^.

After iterated multicollinearity analysis and Pearson’s correlation validation (Supplementary Table 5 and Supplementary Fig. 5), certain preliminary indicators are excluded from consideration as PCI in each region due to their strong associations: PGV, profile curvature, and fault mechanism. PGV represents the integration of PGA over time, and therefore encompasses much of the same information. The topographic variables, profile curvature, and TPI, are highly correlated. While combining fault mechanism with other data can augment the predictive capacity of landslides from a process perspective, it does not offer significant contributions in terms of data information. Hence it was excluded from model construction.

Combined with the ranking and frequency of PCIs in all the regions. We find that PGA, slope, and lithology are most dominant. Relief and roughness are another two important controls. Relief (similar to slope) describes terrain unevenness; it tends to be strongly correlated with slope and appears alternately (with the same average PCI ranking) (Fig. 2). Roughness also performs strongly reflecting surface fluctuation that influence the intensity of erosion processes and hence slope stability. Six indicators (soil type, distance to fault, TPI, land cover, TWI, and plan curvature) are moderate performers; while they contribute to landslide triggering in each region, their impacts are secondary.

Three indicators (distance to river, NDVI, and aspect) are weakest (Fig. 2) among all the PCIs, which may be due to the following reasons. (i) The contribution of the factor is inherently limited. For instance, the role of NDVI in landslide susceptibility mainly reflects in the impact of vegetation cover on the shear strength of slope surface soils. Apparently, this factor is not a predominant one. (ii) They hold less significance on a larger slope than has been previously assumed, being more influential at the local scale. For instance, aspect has shown good performance on landslide susceptibility mapping in the southeast margin of the Qinghai-Tibetan Plateau, since the active faults and mountains close to the epicenters are orientated similarly. The location and orientation of surface ruptures and seismic wave propagation contributed to the distribution of landslides^8^. Moreover, these regions are influenced by the southeast monsoon, and severe weather events can lead to more vulnerable slopes determined by aspect^9^. (iii) Some indicators apparently have limited spatial accuracy. For instance, here river lines were extracted according to catchment topography, not actual river courses, compromising the accuracy of the indicator of distance to river, especially for headwater streams.

Supplementary Note 2. Technical details of deep learning-based model

**Data processing.** The model input consists of multi-channel geospatial images, with each channel representing a specific feature influencing earthquake-triggered landslide. Outliers are clipped to the range of [0, 255] to mitigate noise, and pixel values are then normalized to the range of [0, 1]. For label preprocessing, the Poisson diffusion is applied to smooth the binary landslide labels, thereby reducing noise and emphasizing general spatial patterns^10^. Briefly, given the input binary labels $L$ and the defined background $B$, we solve the Laplace equation on $B$ under Dirichlet boundary conditions. The Laplace operator is discretized using a five-point difference method with iterative updates (Eq. S1-S3).

$$\begin{aligned} \emptyset\left( i,j \right)=L\left( i,j \right) for \left( i,j \right)\notin B\#\left( Eq. S1 \right) \end{aligned}$$

$$\begin{aligned} \nabla^{Z}=\emptyset\left( i,j \right) for \left( i,j \right)\in B\#\left( Eq. S2 \right) \end{aligned}$$

$$\begin{aligned} \emptyset^{k+1}\left( i,j \right)=0.05\times\left[ \emptyset^{k}\left( i+1,j \right)+\emptyset^{k}\left( i-1,j \right)+\emptyset^{k}\left( i,j+1 \right)+\emptyset^{k}\left( i,j-1 \right) \right]\#\left( Eq. S3 \right) \end{aligned}$$

where $L(i,j)\in Z^{H\times W}$ is the landslide binary label field, with $Z$ representing the input matrix (height $H$, width $W$), and $B=\left\{ (i,j)|L\left( i,j \right)=0 \right\}$ is the background area where no landslides are present. $\nabla$ denotes the vector differential operator. $\emptyset$ is the diffusion field vector representing the smoothed labels, with $i$ and $j$ denoting the spatial indices within the matrix $Z$, and $k$ represents the current iteration. The iterative update of $\emptyset$ follows the five-point difference method, where the value at each point is updated based on its neighboring values, allowing for diffusion of the label information across the background region.

To provide a quantitative assessment of our labeling strategy for bias mitigation, we also implemented the conventional binary pixel sampling for comparison. We trained global models with each scheme and performed prediction on two events in data-sparse regions: the 2002 *M*_w_ 7.9 Denali earthquake (Cold CP region) and the 2015 *M*_w_ 7.8 Gorkha earthquake (Cold AH region). Compared to the binary sampling model, the improved sampling model delivered consistent, measurable gains in both scenarios. For the 2002 Denali event, AUC increased by 11.9%, F1 by 16.4%, ACC by 7.9%, and Kappa by 15.9%. Likewise, for the 2015 Gorkha event, we observed AUC + 10.6%, F1 + 10.0%, ACC + 9.3%, and Kappa + 18.7%. These ablation results confirm that our sampling strategy effectively removed event-scale imbalance, especially in underrepresented regions, leading to more accurate and robust predictions.

**Neural network architecture.** We developed a fusion fully convolutional network that incorporates multi-scale attention mechanisms while conforming to differential equation constraints (Eq. S4):

$$\begin{aligned} F\left( I;\theta\right)=D\left( A\left( E\left( I \right)\oplus u\left( E\left( I \right) \right) \right) \right)\#\left( Eq. S4 \right) \end{aligned}$$

where $E$ denotes the encoder, $D$ is the decoder, $A$ represents the multi-scale attention module, and *I* is the input data matrix. $\theta$ represents all learnable weights, $u$ is a parameter-free feature fusion function, and ⊕ represents tensor concatenation. The feature fusion function $u$ combines information from the encoder and attention module to ensure efficient multi-scale feature extraction while avoiding parameter dependence, allowing for a flexible fusion of features without additional learnable parameters.

The encoder follows a progressive feature extraction strategy based on a classic U-Net architecture with four hierarchical levels. Each encoding unit is defined as (Eq. S5):

$$\begin{aligned} Y^{l}=f\left( GELU\left( K^{l}*X^{l} \right) \right)\#\left( Eq. S5 \right) \end{aligned}$$

where $f(\cdot)$ denotes a non-linear transformation, $K^{l}$ is the learnable convolution kernel at level $l$, and $X^{l}$ and $Y^{l}$ are the corresponding input and output matrices at level $l$. The operator $*$ denotes the convolution operation. The Gaussian Error Linear Unit (GELU) activation function is employed in this architecture due to its continuous differentiability, which enhances gradient stability during back-propagation and optimizes the learning process^11^. The feature fusion module utilizes the CBAM, which dynamically captures multi-scale features^12^. The channel attention mechanism, implemented using fully connected layers, extracts high-dimensional features by focusing on relevant landslide-related factors. At the same time, the spatial attention mechanism uses a 7×7 convolutional kernel to capture spatial correlations and dependencies among different features in the two-dimensional space.

The decoder adopts a progressive up-sampling architecture across four levels. Each decoder unit performs bilinear up-sampling, with skip connections facilitating the transfer of high-frequency detail features through learnable deconvolution kernels. This design refines the prediction and ensures that fine-grained spatial details are preserved throughout the network. The decoder's architecture is defined as (Eq. S6):

$$\begin{aligned} Y^{l}=f\left( GELU\left( K_{d}^{l}*X^{l} \right)\oplus\varepsilon^{l-1} \right)\#\left( Eq. S6 \right) \end{aligned}$$

where $K_{d}^{l}$ is the learnable deconvolution kernel at level $l$, $X^{l}$ and $Y^{l}$ are the corresponding input and output matrices, $\varepsilon^{l-1}$ is the output from the encoder at level$l-1$, and ⊕ represents tensor concatenation. The deconvolution operation restores high-resolution features from lower-resolution maps, facilitated by skip connections from the encoder to maintain critical spatial information.

The above architecture allows the model to efficiently process multi-scale features, capture both high- and low-level spatial relationships, and produce accurate predictions for earthquake-triggered landslides.

**Hyperparameter settings.** The model was trained with the Adam optimizer under the following hyperparameters: an initial learning rate of 0.0001, a batch size of 16, and 100 training epochs. To ensure stable convergence, we implemented a learning rate decay strategy—reducing the rate by a factor of 0.5 if the validation loss does not improve after five epochs. To mitigate overfitting, early stopping was applied with a patience of 20 epochs. Training is halted if no improvement is observed over 20 consecutive epochs. In addition, if the validation loss plateaus for three consecutive epochs, the learning rate is further halved. Model performance was evaluated by mean squared error (MSE) loss function, which collectively assess the predictive power of the landslide susceptibility.

Supplementary Note 3. Landslide prediction for an independent event within each region

To minimize the impact of data uncertainty on predictive performance in the five independent testing, we computed the mean probability of landslide occurrence from 10 Monte Carlo training-prediction iterations. This strategy helps filter out high-uncertainty predictions and reduces model performance fluctuations caused by data randomness in each sampling, thereby enhancing the reliability of the results and subsequent comparative analyses.

In the equatorial CP region, the predictive map of the global model for the 2021 Nippes event bears a remarkable resemblance to that of the regional model at any given location, albeit with an overall higher susceptibility (+7.3%, note that landslide thresholds increase with growing probabilities), resulting in approximately equal accuracies (best among all five cases). Despite the epicenter and most violent shaking being located in the northeast plain, the landslides were concentrated in the northwest mountains of Pic Macaya National Park and the hilly southeast areas with steep topography. These locations are highly consistent with the predictions of both models. We compared three aspects of the models (as with all five earthquake events analysed in this section), including the richness of training samples, the PCIs, and their ranking. In addition to the 2021 Nippes earthquake, the equatorial CP belt offers six independent events (distributed in the coastal zones of South America and Oceania) with sufficient data (59,423 landslides) for training. Compared to the global model, the equatorial CP model considered one additional PCI (relief), and the PCI ranking does not exhibit significant differences.

For the case of the 2016 Kaikoura earthquake, both global and regional models performed well (global AUC = 87.5% and regional AUC = 88.8%) with sufficient training samples supported (46,735 landslides in ten events within the temperate CP region). Their predictions are highly consistent with landslide distribution, which generally follow the spread of strong seismic intensity. Considering the PCIs, the most notable change with the regional model is the promotion of the importance of lithology, moving from third to first place. Accordingly, we found that the variance of predicted landslide areas among lithologies based on the regional model is higher than that of the global model.

Regarding the temperate AH region through the test case of the 2022 Luding earthquake, both global and regional models achieved good predictions (AUC = 84.1% and 85.0%, respectively) thanks to abundant training events (thirteen) and samples (229,862 landslides). The global model produces a significant higher overall probability (+18.94%), which demonstrates our model performance in a region extremely vulnerable to landslides: the southeast margin of the Qinghai-Tibetan Plateau. Relative to the global model, the PCI rankings of distance to river and fault on the regional scale improved notably, and the corresponding performance shows that the detected landslide areas close to rivers (within 1 km) and faults (within 5 km) in the regional model are significantly broader (+88.61% and +36.42%). Within this region, due to long-term tectonic uplift and river incision, rocks near the active faults are densely fractured, resulting in unstable adjacent slopes under seismic forces^13^. The enhanced performance of regional model is attributed to the adequate learning and attention to these local characteristics.

Turning to the cases located in cold CP and AH regions, the regional models underperformed relative to the global model. The outcomes of the 2015 Gorkha event (Cold AH region) turned out to be less favorable with AUC of 77.26% (global model) and 76.74% (regional model). This region includes only two training events (2013 Minxian and 2017 Mainling events), resulting in a small sample diversity for model learning (3,779 landslides). The PCI content and ranking of the two models are similar, where the slope in the global model is replaced by the relief indicator in the regional model. What has changed significantly is the increasing importance of distance to fault. The corresponding predictions show that the landslide area close to faults (within 5 km) detected by the regional model is 5.16 km^2^ larger than that of the global model. However, the actual landslides appear far from known active faults in this event, suggesting some overfitting bias as well as the inferior performance of the regional model.

With consideration of different scales, the model predictions have some distinguishing emphasis in the 2002 Denali event of the Cold CP region. Due to the scarcity of training events within this region (2007 Aysén Fjord and 2018 Hokkaido events), the training samples for the regional model are not sufficient and appear unbalanced (8,500 landslides). In terms of PCI, the regional model has four fewer factors compared to the global one, and (typically) strong performers, PGA and lithology, are absent. Accordingly, the predicted landslide area based on the global model is 15.74% larger than that of the regional model within high seismic intensity (PGA > 0.3 g). Meanwhile, the variance of predicted landslide areas among lithologies of the global model is also higher than that of the regional model. These major differences are due to insufficient training of the regional model, limiting its accuracy, AUC of 71.13% compared to AUC of 73.38% for the global model.

As mentioned above, the performance of regional models is superior in most scenarios, as they are capable of capturing the local characteristics of landslide development under similar geological settings and climatic conditions. However, regional model outcomes are not always optimal in certain circumstances, due to insufficient or imbalanced training. This leads to inadequate learning of the distribution patterns of landslides developed under unique backgrounds and the potential for overfitting models in favor of events with more landslides. In contrast, the global model considers the development patterns of landslides in widely different environments and with sufficient training samples. Therefore, we suggest a strategy, which adopts regional models in the equatorial CP, AH, and temperate AH regions (with abundant and diverse events), whereas the global model is superior for the Cold CP and AH regions (owing to fewer training events).

Supplementary Note 4. Model sensitivity to data uncertainty

To quantify how input uncertainties propagate through our framework, we performed two sensitivity experiments using the 2016 Kaikoura *M*_w_ 7.8 earthquake as an example:

**PGA-uncertainty sensitivity.** We obtained the USGS ShakeMap PGA and its accompanying pixel-wise uncertainty estimates. Ten perturbed PGA scenarios were generated by overlaying a 5% random sampled uncertainty (Monte Carlo sampling). Holding all other model inputs constant, we ran our landslide prediction on each scenario and computed performance metrics. Across these ten experimental runs, the 95% confidence intervals were: AUC = 88.82% ± 0.02%, F1 = 80.92% ± 0.06%, ACC = 80.49% ± 0.03%, and Kappa = 61.08% ± 0.05%.

**Label-uncertainty sensitivity.** The boundary of landslides is often the most common interpretation error due to the coarse resolution of remote sensing images and the perceived differences in identification. To simulate these common boundary-interpretation errors, we shifted the binary landslide labels for all coseismic inventories in the Temperate CP region by one pixel in each cardinal direction (up, down, left, right, upper-left, upper-right, lower-left, and lower-right), retraining the model separately on each of these biased inventories. We then predicted the 2016 Kaikoura earthquake-triggered landslides with each retrained model as well as the original one. The resulting 95% confidence intervals were: AUC = 88.80% ± 0.08%, F1 = 80.83% ± 0.22%, ACC = 80.45% ± 0.07%, and Kappa = 61.02% ± 0.07%.

These experiments offer a systematic and quantitative evaluation of how uncertainties in data inputs influence deep-learning predictions. Their narrow confidence intervals indicate that our models are robust to realistic perturbations in PGA and errors in landslide interpretation.

Supplementary Note 5. Hypothetical earthquake simulation

The Anninghe Fault is a large left-lateral strike-slip fault along the eastern boundary of the Tibetan Plateau, a region characterized by high seismic activity. Historical records indicate that the fault has experienced numerous strong earthquakes with magnitudes of *M*_w_ ≥ 7.0. In this study, we conducted simulations of broadband strong ground motion for a scenario earthquake with a magnitude of *M*_w_ 7.5, within a box-like volume measuring 310 km by 230 km at the surface and extending to 28 km in depth. Our simulations incorporate a three-dimensional (3D) Earth structure, finite-fault rupture, and realistic surface topography. We applied a hybrid broadband method to simulate ground motions within the frequency range of 0.1–20 Hz.

The kinematic stochastic source rupture was generated using the GP method^14, 15^, which incorporates random distributions of slip, rupture velocity, and rise time on the rupture surface. Based on the geological conditions and kinematic characteristics of the fault zone, the strike, dip, and slip angles of the seismic rupture plane were set to 180°, 70°, and 15°, respectively. Historically, the Mianning–Xichang segment of the Anninghe Fault Zone has experienced frequent strong earthquakes, so the epicenter is positioned within this segment. The geometric dimensions of the earthquake rupture surface were determined using the empirical relationships for strike-slip earthquakes^16^. Based on historical seismic data compiled within a 150 km radius around Xichang, the average focal depth in this region is approximately 18 km. Therefore, we set the focal depth of the simulated earthquake to 18 km.

The low-frequency seismograms (<1 Hz) were simulated using a 3D curvilinear grid finite-difference algorithm^17, 18^, which employs boundary-conforming grids to accurately represent the irregular free surface. In this algorithm, the spatial finite-difference operator is implemented using the DRP/opt MacCormack scheme, achieving fourth-order accuracy in dispersion by optimizing numerical dispersion and dissipation errors. The time marching scheme is based on the fourth-order Runge-Kutta method. Topographic elevations were extracted from ALOS AW3D30. We utilized a 3D high-resolution Vp and Vs model for the crust and uppermost mantle of southwest China (SWChinaCVM-2.0)^19^. The medium was discretized with a horizontal grid spacing of 300 m, while the vertical grid spacing increases progressively from 50 to 200 m, resulting in a total grid count of 994 × 894 × 227.

High-frequency seismograms were calculated using EXISM^20^. The empirical site amplification model employs the amplification effects corresponding to the site conditions at the B-C boundary as defined by the National Earthquake Hazards Reduction Program (NEHRP) in the United States^21^. Nonlinear site amplification factors related to the period were computed using a VS30 prediction model tailored for the Chinese region based on topographic characteristics. The high-frequency attenuation parameter, associated with site effects, was set to 0.035 s.

Subsequently, we combined the high- and low-frequency seismograms to obtain broadband seismic motion by aligning the acceleration time histories with respect to the S-wave arrival time. These aligned time histories were then transformed into the frequency domain. The low-frequency and high-frequency Fourier spectra were filtered separately using low-pass and high-pass filters with matching cutoff frequencies, and finally, the filtered spectra were superimposed to generate broadband ground motions. Supplementary Fig. 6 displays the PGA and seismic intensity distributions.


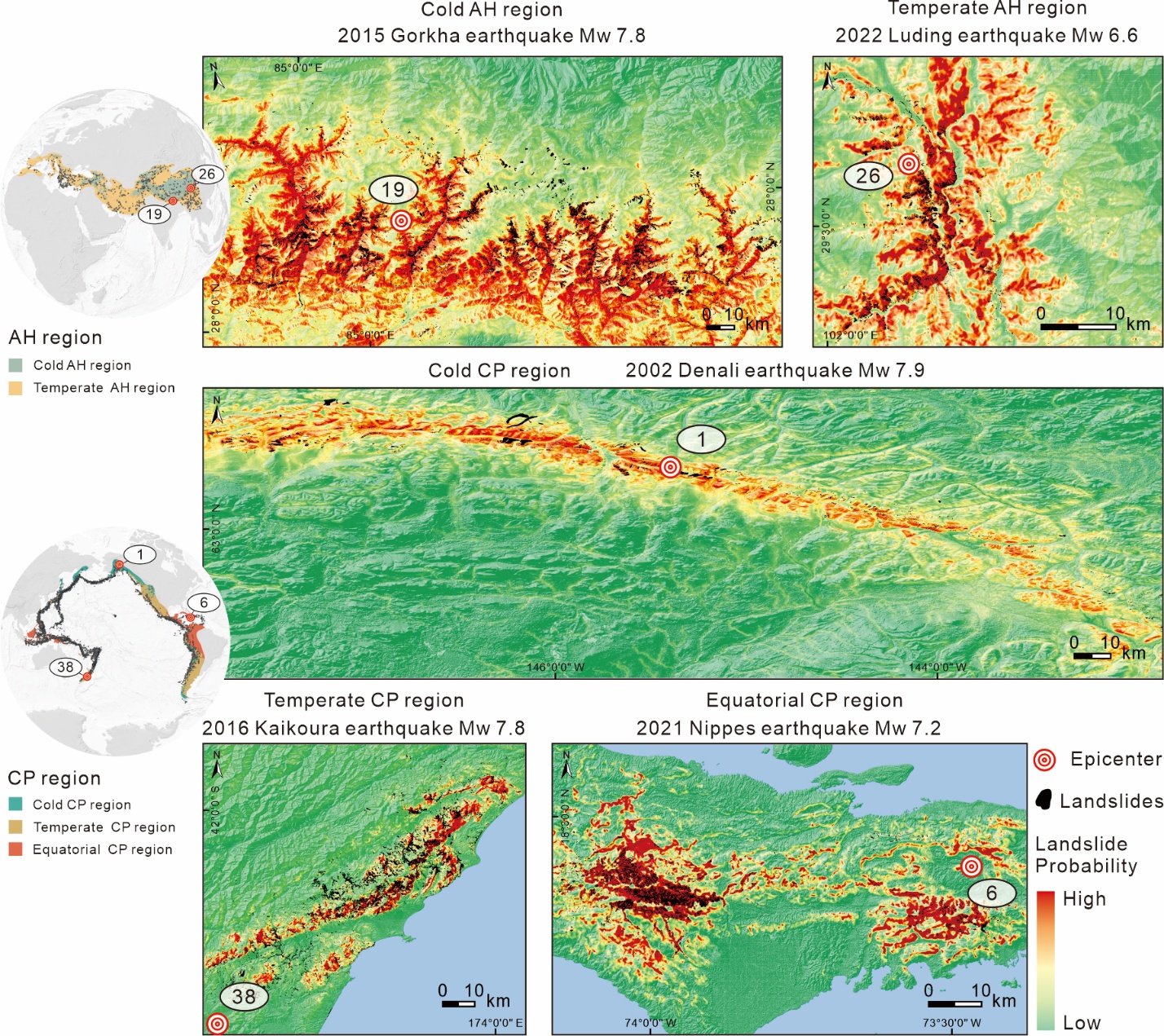


Supplementary Fig. 1. Landslide predictions of the underperformed models for five major earthquake events.


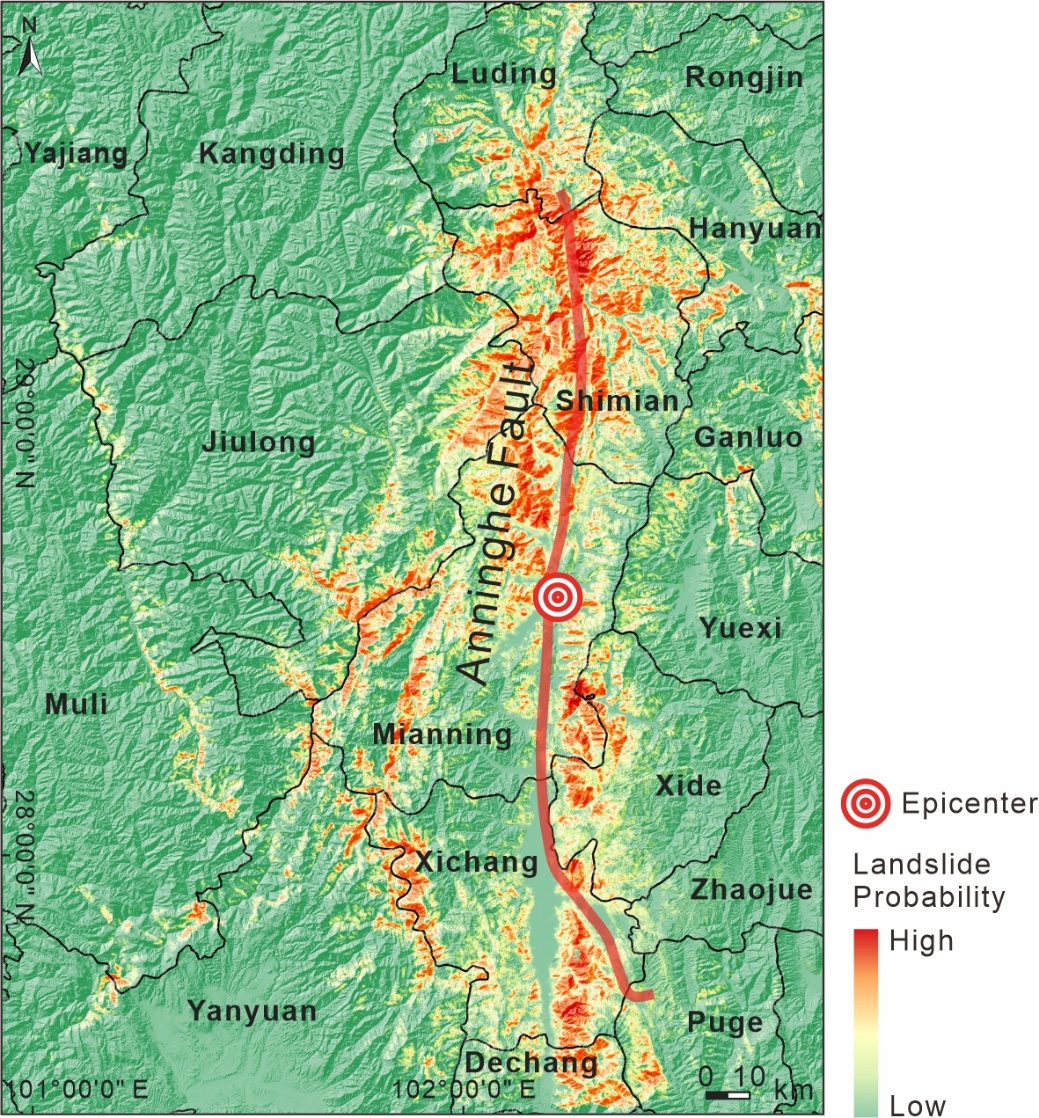


Supplementary Fig. 2. Landslide prediction of the hypothetical scenario.


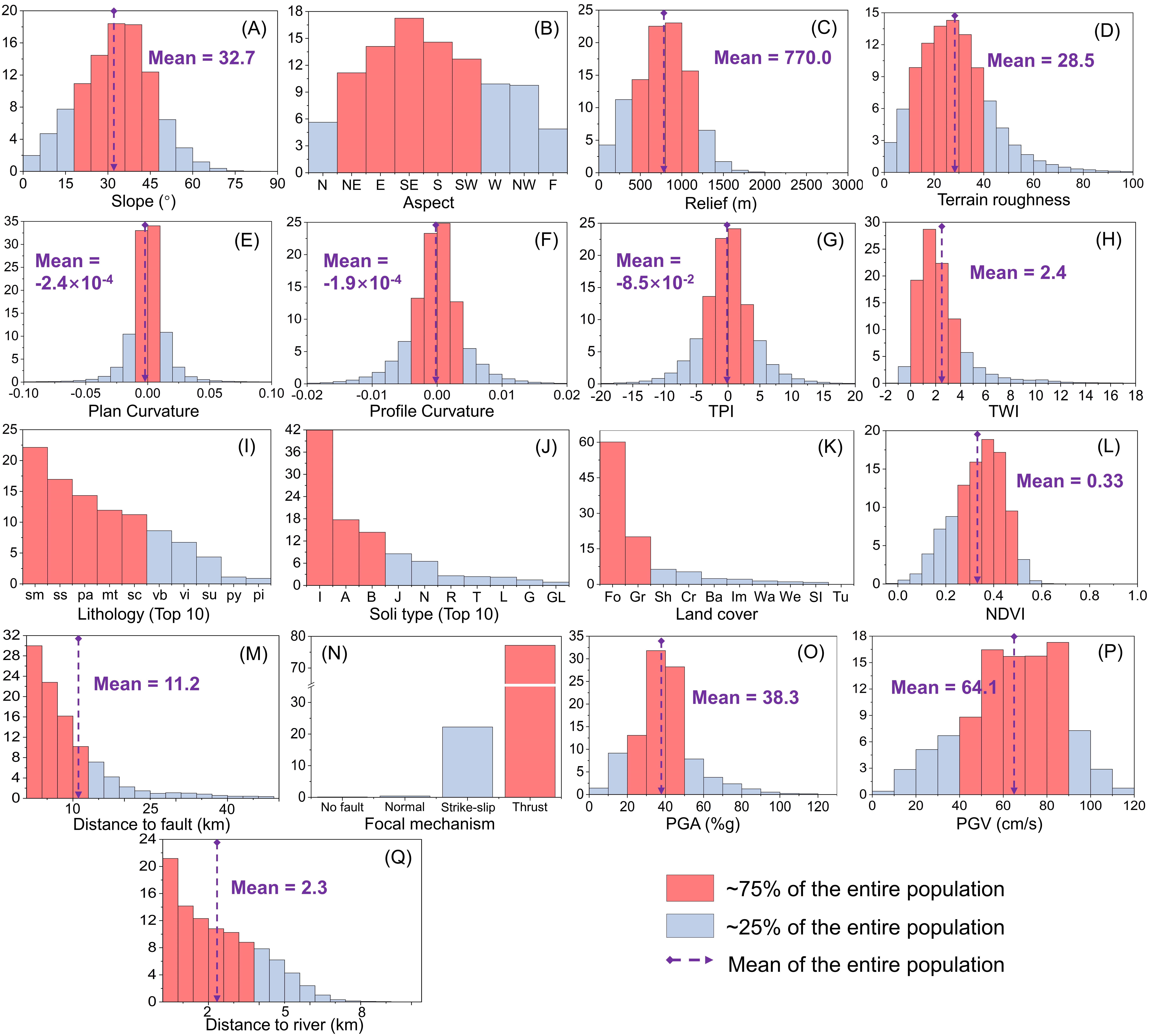


Supplementary Fig. 3. Global patterns of landslide distribution under various conditioning factors of (A) slope, (B) aspect, (C) relief, (D) terrain roughness, (E) plan curvature, (F) profile curvature, (G) TPI, (H) TWI, (I) lithology, (J) soil type, (K) land cover, (L) NDVI, (M) distance to fault, (N) focal mechanism, (O) PGA, (P) PGV, and (Q) distance to river. The soil type abbreviations: I = lithosols, A = acrisols, B = cambisols, J = fluvisols, N = nitosols, R = regosols, T = andosols, L = luvisols, and G = gleysols; The lithology abbreviations: sm = mixed sedimentary rocks, ss = siliciclastic sedimentary rocks, pa = acid plutonic rocks, mt = metamorphics, sc = carbonate sedimentary rocks, vb = basic volcanic rocks, vi = intermediate volcanic rocks, su = unconsolidated sediments, py = pyroclastics, and pi = intermediate plutonic rocks; The land cover abbreviations: Fo = forest, Gr = grassland, Sh = shrubland, Cr = cropland, Ba = bare, Im = impervious surface, Wa = water, We = wetland, SI = snow/ice, and Tu = tundra.


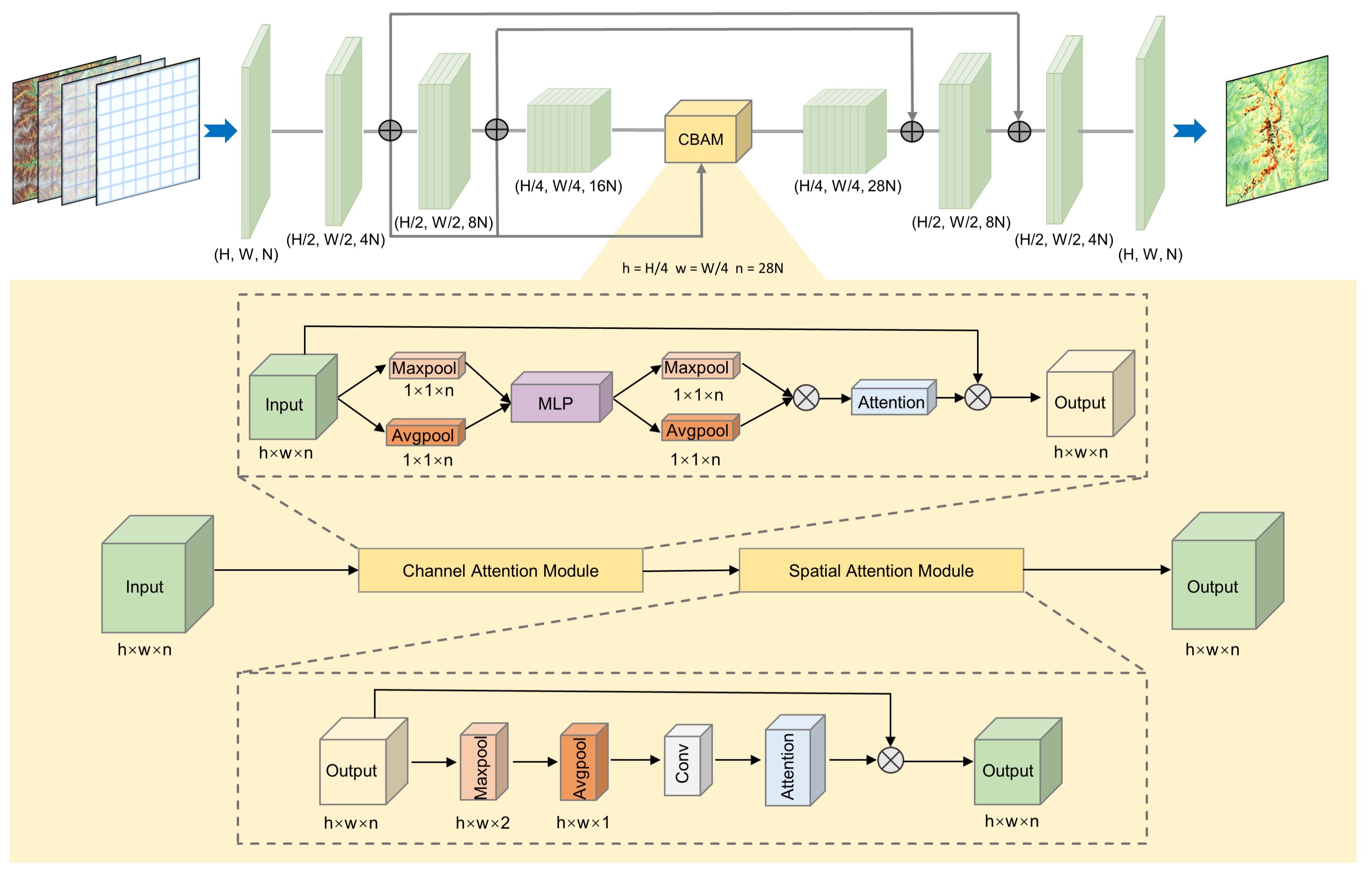


Supplementary Fig. 4. Structure of the proposed FCN-based model for landslide prediction.


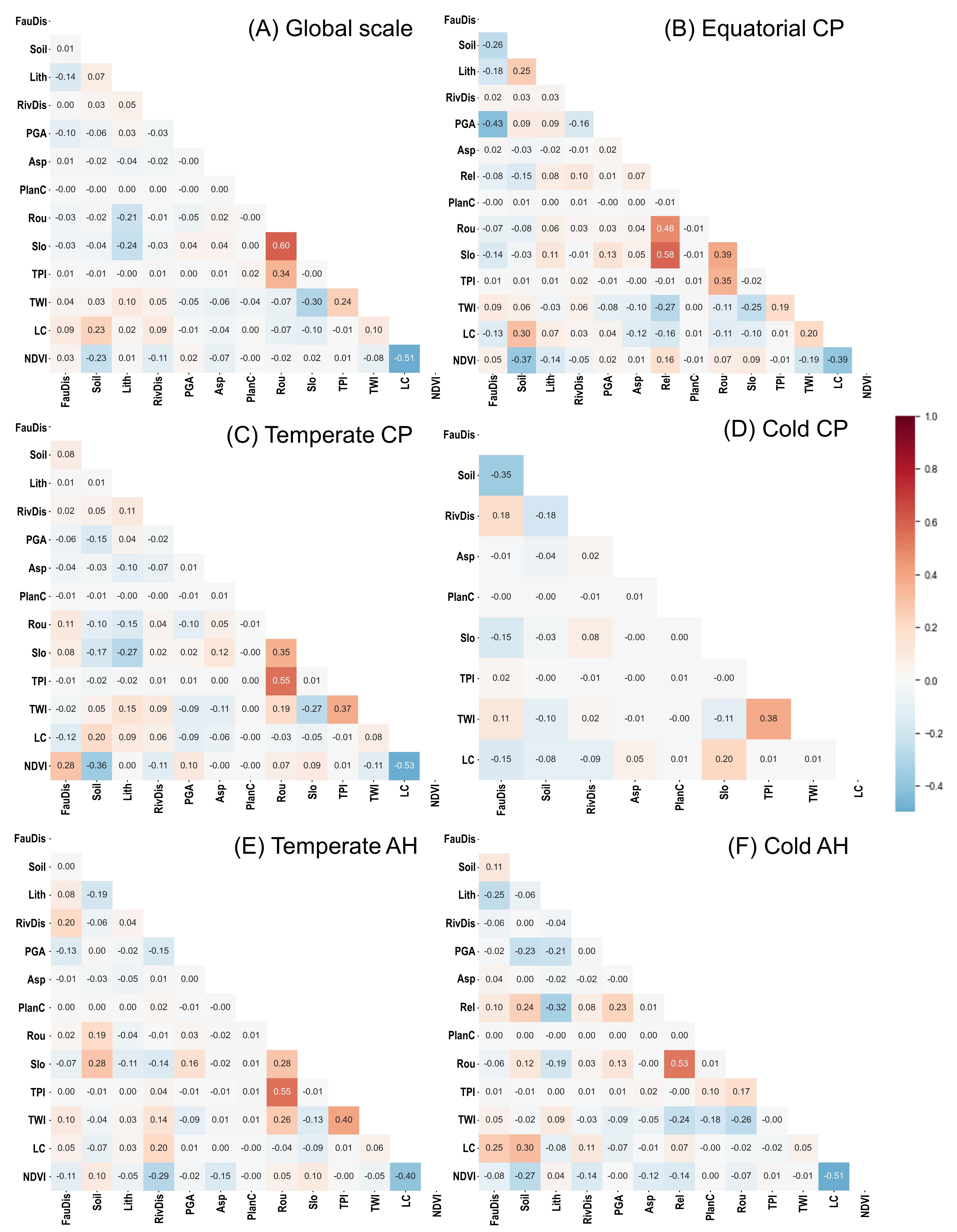


Supplementary Fig. 5. Pearson’s analysis results of the final PCIs among (A) global scale, (B) equatorial CP, (C) temperate CP, (D) cold CP, (E) temperate AH, and (F) cold AH regions. The indicator abbreviations: FauDis = distance to fault, Soil = soil type, Lith = lithology, RivDis = distance to river, Asp = aspect, Rel: relief, PlanC = plan curvature, Rou = terrain roughness, Slo = slope, and LC = land cover.


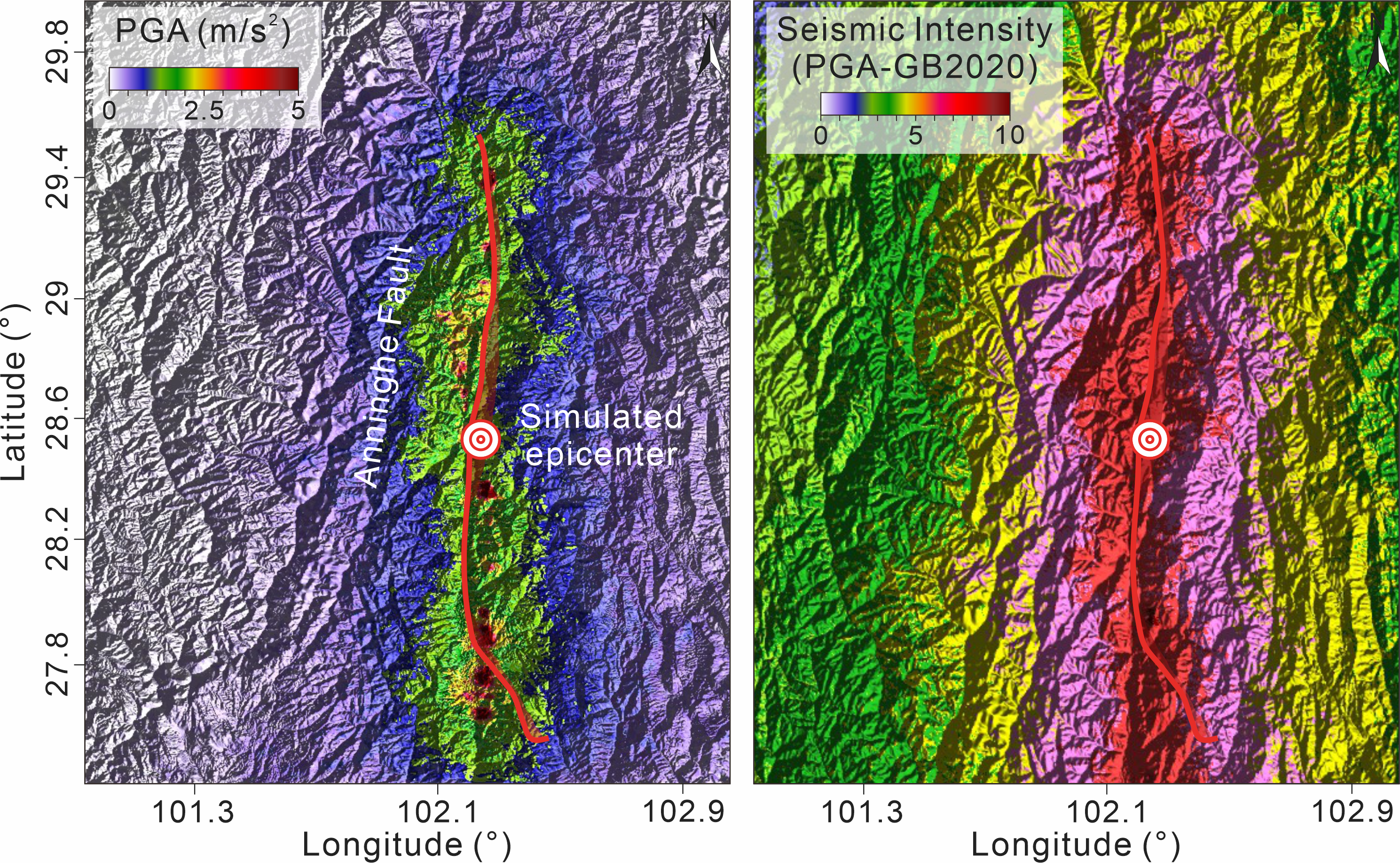


Supplementary Fig. 6. Distribution of the simulated PGA and seismic intensity.

Supplementary Table 1. Detailed summary of global earthquake-triggered landslide inventories.

| ID | Event | Country | Latitude | Longitude | Time | *M*_w_ | Landslides |
| --- | --- | --- | --- | --- | --- | --- | --- |
| 8 | Guatemala City^22^ | Guatemala | 15.32°N | 89.10°W | 1976-02-04 | 7.5 | 6224 |
| 3 | Coalinga^23^ | USA | 36.23°N | 120.31°W | 1983-05-02 | 6.7 | 3980 |
| 2 | Loma Prieta^24^ | USA | 37.04°N | 121.88°W | 1989-10-18 | 6.9 | 1775 |
| 9 | Limon^25^ | Costa Rica | 9.69°N | 83.07°W | 1991-04-22 | 7.7 | 1643 |
| 4 | Northridge^26^ | USA | 34.21°N | 118.54°W | 1994-01-24 | 6.7 | 11111 |
| 32 | Great Hanshin^27^ | Japan | 34.59°N | 135.07°E | 1995-01-16 | 6.9 | 2353 |
| 12 | Umbria-Marche^28^ | Italy | 43.08°N | 12.81°E | 1997-09-26 | 6.0 | 179 |
| 34 | Jueili^29^ | China | 23.50°N | 120.70°E | 1998-07-17 | 5.7 | 847 |
| 33 | Chi-Chi^30^ | China | 23.77°N | 120.98°E | 1999-09-21 | 7.6 | 13783 |
| 1 | Denali^31^ | USA | 63.51°N | 147.60°W | 2002-11-03 | 7.9 | 1579 |
| 17 | Lefkada Island^32^ | Greece | 38.79°N | 20.56°E | 2003-08-14 | 6.3 | 274 |
| 31 | Chuetsu^33^ | Japan | 37.30°N | 138.80°E | 2004-10-23 | 6.6 | 4862 |
| 18 | Kashmir^34^ | Pakistan | 34.45°N | 73.65°E | 2005-10-08 | 7.6 | 2930 |
| 5 | Kiholo Bay^35^ | USA | 19.88°N | 155.94°W | 2006-10-15 | 6.7 | 383 |
| 10 | Aysén Fjord^36^ | Chile | 45.27°S | 72.66°W | 2007-04-21 | 6.2 | 538 |
| 22 | Wenchuan^37^ | China | 31.02°N | 103.37°E | 2008-05-12 | 7.9 | 197481 |
| 29 | Iwate^38^ | Japan | 39.03°N | 140.88°E | 2008-06-13 | 6.9 | 4164 |
| 16 | L'Aquila^39^ | Italy | 42.35°N | 13.38°E | 2009-04-06 | 6.3 | 94 |
| 7 | Port-au-Prince^40^ | Haiti | 18°28′N | 72°32′W | 2010-01-12 | 7.0 | 23567 |
| 30 | Tōhoku^41^ | Japan | 38.32°N | 142.37°E | 2011-03-11 | 9.1 | 3477 |
| 11 | Lorca^42^ | Spain | 37.70°N | 1.67°W | 2011-05-11 | 5.1 | 258 |
| 24 | Lushan^43^ | China | 30.37°N | 102.94°E | 2013-04-20 | 6.6 | 22820 |
| 21 | Minxian^44^ | China | 34.50°N | 104.20°E | 2013-07-21 | 5.9 | 2330 |
| 27 | Ludian^45^ | China | 27.25°N | 103.43°E | 2014-08-03 | 6.2 | 1024 |
| 19 | Gorkha^46^ | Nepal | 28.23°N | 84.73°E | 2015-04-25 | 7.8 | 21151 |
| 15 | Central Italy 1^47^ | Italy | 42.71°N | 13.22°E | 2016-08-24 | 6.2 | 145 |
| 13 | Central Italy 2^47^ | Italy | 42.86°N | 13.09°E | 2016-10-26 | 6.1 | 235 |
| 14 | Central Italy 3^47^ | Italy | 42.86°N | 13.09°E | 2016-10-30 | 6.6 | 378 |
| 38 | Kaikoura^48^ | New Zealand | 42.76°S | 173.08°E | 2016-11-14 | 7.8 | 14233 |
| 23 | Jiuzhaigou^49^ | China | 33.19°N | 103.86°E | 2017-08-08 | 6.5 | 2895 |
| 20 | Mainling^50^ | China | 29.87°N | 95.02°E | 2017-11-17 | 6.4 | 1449 |
| 36 | Hela^51^ | Papua New Guinea | 6.07°S | 142.75°E | 2018-02-25 | 7.5 | 11607 |
| 37 | Lombok^52^ | Indonesia | 8.29°S | 116.45°E | 2018-08-19 | 6.9 | 9319 |
| 28 | Hokkaido^53^ | Japan | 42.67°N | 141.93°E | 2018-09-06 | 6.6 | 7962 |
| 35 | Palu^54^ | Indonesia | 0.18°S | 119.84°E | 2018-09-28 | 7.5 | 7063 |
| 6 | Nippes^55^ | Haiti | 18.42°N | 73.48°W | 2021-08-14 | 7.2 | 7889 |
| 25 | Lushan^56^ | China | 30.42°N | 102.99°E | 2022-06-01 | 5.9 | 1149 |
| 26 | Luding^57^ | China | 29.49°N | 102.22°E | 2022-09-05 | 6.6 | 5547 |

Supplementary Table 2. Performance of landslide predictions in the cases for each region.

| Index | Equatorial CP | | | Temperate CP | | | Cold CP | | | Temperate AH | | | Cold AH | | |
| --- | --- | --- | --- | --- | --- | --- | --- | --- | --- | --- | --- | --- | --- | --- | --- |
|  | GM^*^ | | RM^†^ | GM | | RM | GM | | RM | GM | | RM | GM | | RM |
| AUC | 94.9 | | 94.9 | 87.5 | | 88.8 | 73.4 | | 71.1 | 84.1 | | 88.5 | 77.3 | | 76.7 |
| Precision | 86.6 | | 86.6 | 76.6 | | 77.6 | 67.3 | | 65.5 | 75.9 | | 80.3 | 75.8 | | 75.0 |
| Recall | 90.1 | | 90.6 | 85.5 | | 85.8 | 69.6 | | 68.3 | 80.4 | | 76.1 | 68.6 | | 64.0 |
| F1 | 88.3 | | 88.6 | 80.8 | | 81.5 | 68.4 | | 66.8 | 78.1 | | 81.8 | 72.0 | | 69.1 |
| ACC | 88.1 | | 88.3 | 79.7 | | 80.5 | 67.9 | | 66.1 | 77.5 | | 81.5 | 73.3 | | 71.3 |
| Kappa | 76.2 | | 76.6 | 59.4 | | 61.0 | 35.8 | | 32.2 | 54.9 | | 63.0 | 46.6 | | 42.7 |
| Area | 9645 km^2^ | | | 28111 km^2^ | | | 92316 km^2^ | | | 2810 km^2^ | | | 56468 km^2^ | | |
| Training^‡^ | 104.6 h | | 29.1 h | 102.9 h | | 28.0 h | 97.8 h | | 6.4 h | 105.1 h | | 37.2 h | 100.9 h | | 8.0 h |
| Prediction | 4 s | 4 s | | 13 s | 13 s | | 51 s | 51 s | | 1 s | 1 s | | 24 s | 24 s | |

^*^ Global model accuracy (%). ^†^ Regional model accuracy (%).

^‡^ The landslide prediction framework was developed using Python 3.7.0 with TensorFlow-GPU 2.7.0, deployed on a Windows 10 OS powered by dual Tesla V100 GPUs, an Intel Xeon W-2255 CPU, and a 256GB RAM.

Supplementary Table 3. Performance of landslide predictions for all the 38 cases by leave-one-out cross validation.

| Event | AUC | AUC | Pre^‡^ | Pre | Rec^§^ | Rec | F1 | F1 | ACC | ACC | Kappa | Kappa |
| --- | --- | --- | --- | --- | --- | --- | --- | --- | --- | --- | --- | --- |
| No. | GM^*^ | RM^†^ | GM | RM | GM | RM | GM | RM | GM | RM | GM | RM |
| 8 | 77.75 | 81.96 | 67.86 | 74.38 | 78.82 | 75.79 | 72.93 | 75.08 | 70.74 | 74.84 | 41.48 | 49.69 |
| 3 | 83.07 | 83.08 | 75.63 | 75.69 | 78.47 | 78.43 | 77.02 | 77.04 | 76.59 | 76.62 | 53.19 | 53.24 |
| 2 | 81.10 | 79.61 | 74.56 | 71.99 | 78.97 | 81.39 | 76.70 | 76.40 | 76.01 | 74.86 | 52.03 | 49.72 |
| 9 | 76.21 | 87.86 | 67.24 | 78.21 | 72.28 | 85.37 | 69.67 | 81.63 | 68.54 | 80.79 | 37.07 | 61.58 |
| 4 | 93.61 | 93.97 | 86.87 | 86.23 | 87.88 | 90.66 | 87.37 | 88.39 | 87.30 | 88.09 | 74.59 | 76.18 |
| 32 | 87.15 | 88.33 | 78.26 | 85.23 | 89.11 | 74.26 | 83.33 | 79.37 | 82.18 | 80.69 | 64.36 | 61.39 |
| 12 | 86.70 | 84.49 | 78.01 | 78.33 | 85.44 | 74.22 | 81.56 | 76.22 | 80.68 | 76.84 | 61.36 | 53.69 |
| 34 | 62.66 | 65.48 | 61.55 | 60.23 | 53.38 | 68.22 | 57.17 | 63.98 | 60.02 | 61.59 | 20.03 | 23.18 |
| 33 | 91.82 | 91.27 | 82.60 | 81.63 | 87.53 | 88.78 | 84.99 | 85.05 | 84.54 | 84.40 | 69.08 | 68.80 |
| 1 | 73.38 | 71.13 | 67.33 | 65.45 | 69.59 | 68.27 | 68.44 | 66.83 | 67.91 | 66.12 | 35.82 | 32.23 |
| 17 | 86.33 | 90.44 | 77.34 | 87.15 | 79.51 | 72.71 | 78.41 | 79.28 | 78.10 | 81.00 | 56.21 | 61.99 |
| 31 | 69.62 | 77.37 | 62.45 | 67.68 | 78.92 | 78.11 | 69.73 | 72.52 | 65.74 | 70.40 | 31.48 | 40.80 |
| 18 | 72.74 | 73.61 | 64.72 | 66.45 | 73.51 | 72.18 | 68.83 | 69.19 | 66.72 | 67.87 | 33.44 | 35.73 |
| 5 | 96.21 | 97.08 | 91.12 | 92.07 | 93.66 | 98.18 | 92.38 | 95.02 | 92.27 | 94.86 | 84.54 | 89.71 |
| 10 | 81.54 | 80.22 | 71.80 | 68.77 | 77.27 | 80.95 | 74.43 | 74.37 | 73.46 | 72.10 | 46.92 | 44.20 |
| 22 | 88.28 | 91.78 | 78.03 | 81.76 | 92.32 | 89.96 | 84.58 | 85.67 | 83.16 | 84.95 | 66.33 | 69.89 |
| 29 | 81.67 | 82.36 | 71.74 | 73.02 | 79.64 | 78.43 | 75.48 | 75.62 | 74.13 | 74.72 | 48.27 | 49.44 |
| 16 | 66.04 | 66.18 | 61.44 | 65.57 | 75.55 | 53.59 | 67.77 | 58.98 | 64.07 | 62.72 | 28.14 | 25.45 |
| 7 | 85.30 | 85.80 | 72.35 | 74.98 | 88.23 | 83.36 | 79.50 | 78.95 | 77.26 | 77.77 | 54.51 | 55.54 |
| 30 | 76.78 | 76.69 | 68.49 | 68.99 | 94.08 | 96.74 | 79.27 | 80.55 | 75.40 | 76.63 | 50.80 | 53.27 |
| 11 | 68.64 | 82.45 | 72.58 | 71.18 | 50.09 | 85.96 | 59.27 | 77.88 | 65.58 | 75.58 | 31.17 | 51.17 |
| 24 | 76.66 | 79.53 | 69.78 | 69.72 | 74.87 | 80.45 | 72.24 | 74.70 | 71.23 | 72.75 | 42.45 | 45.51 |
| 21 | 86.46 | 69.29 | 84.42 | 68.67 | 79.81 | 54.16 | 82.05 | 60.56 | 82.54 | 64.73 | 65.08 | 29.45 |
| 27 | 81.83 | 82.17 | 71.20 | 73.85 | 80.82 | 74.74 | 75.70 | 74.29 | 74.06 | 74.14 | 48.12 | 48.28 |
| 19 | 77.26 | 76.74 | 75.76 | 75.01 | 68.56 | 63.98 | 71.98 | 69.06 | 73.31 | 71.33 | 46.63 | 42.67 |
| 15 | 71.15 | 77.67 | 65.72 | 68.38 | 76.25 | 83.39 | 70.60 | 75.14 | 68.24 | 72.41 | 36.48 | 44.82 |
| 13 | 83.72 | 84.42 | 73.13 | 78.09 | 82.38 | 82.38 | 77.48 | 79.13 | 76.06 | 78.85 | 52.11 | 57.70 |
| 14 | 78.33 | 83.10 | 68.95 | 71.73 | 79.17 | 89.23 | 73.71 | 79.53 | 71.76 | 77.03 | 43.51 | 54.07 |
| 38 | 87.47 | 88.75 | 76.60 | 77.60 | 85.54 | 85.80 | 80.82 | 81.49 | 79.70 | 80.51 | 59.40 | 61.03 |
| 23 | 88.18 | 88.33 | 79.35 | 78.99 | 83.85 | 86.36 | 81.54 | 82.51 | 81.01 | 81.70 | 62.02 | 63.39 |
| 20 | 87.62 | 86.86 | 78.23 | 76.34 | 84.00 | 82.75 | 81.01 | 79.42 | 80.31 | 78.55 | 60.63 | 57.10 |
| 36 | 80.06 | 81.52 | 70.59 | 70.21 | 85.80 | 87.83 | 77.46 | 78.04 | 75.03 | 75.29 | 50.06 | 50.58 |
| 37 | 84.13 | 83.43 | 74.97 | 74.42 | 79.98 | 79.13 | 77.39 | 76.70 | 76.64 | 75.96 | 53.28 | 51.92 |
| 28 | 69.56 | 60.65 | 64.70 | 59.66 | 83.04 | 67.50 | 70.17 | 62.59 | 64.70 | 59.66 | 29.40 | 19.32 |
| 35 | 87.97 | 91.23 | 78.73 | 81.63 | 79.45 | 87.01 | 79.09 | 84.23 | 78.99 | 83.71 | 57.99 | 67.43 |
| 6 | 94.87 | 94.89 | 86.60 | 86.57 | 90.12 | 90.62 | 88.33 | 88.55 | 88.09 | 88.28 | 76.18 | 76.56 |
| 25 | 81.44 | 86.37 | 70.91 | 76.39 | 85.54 | 83.19 | 77.54 | 79.65 | 75.23 | 78.74 | 50.45 | 57.48 |
| 26 | 84.05 | 88.50 | 75.93 | 80.30 | 80.42 | 76.10 | 78.11 | 81.80 | 77.46 | 81.50 | 54.92 | 63.00 |

^*^ Global model accuracy (%). ^†^ Regional model accuracy (%).

^‡^ Precision (%). ^§^ Recall (%).

Supplementary Table 4. General conditioning factors of earthquake-triggered landslides.

| Category | Factor | Description | Data source | Resolution  (Scale) |
| --- | --- | --- | --- | --- |
| Topography | Slope | Steepness of land surface. | ALOS AW3D30  DEM | 30 m |
|  | Aspect | Direction of downhill slope faces. |  |  |
|  | Relief | Topography fluctuation of slope. |  |  |
|  | TPI | Difference between each cell slope value to the mean slope of the cell's neighbors to determine ruggedness of terrain. |  |  |
|  | Terrain roughness | Change in surface fluctuation which reflects surface erosion degree. |  |  |
|  | Plan curvature | Perpendicular to the slope and affects the convergence and divergence of flow across the surface. |  |  |
|  | Profile curvature | Parallel to the slope and indicates the direction of maximum slope. |  |  |
| Geo-ecology | Lithology | Rock material composition in landslide development. | GLiM | 1:3750000 |
|  | Soil type | Material mechanism of landslide mass. | FAO | 1:5000000 |
|  | Land cover | Terrain environment in landslide development. | GLC_FCS30D | 30 m |
|  | NDVI | Density of vegetation greenness on landslide slope. | Landsat 4-8 |  |
| Hydrology | TWI | Describing the tendency of runoff flow direction and accumulation of landslide slope. | ALOS AW3D30 DEM | 30 m |
|  | Distance to river | Erosion extends of landslide slope. |  |  |
| Seismology | Distance to fault | Shaking extents of landslide slope. | GEM & CAFD | 1:40000 |
|  | Focal mechanism | Direction of slip in an earthquake and the orientation of the fault on which it occurs. |  |  |
|  | PGA | Maximum ground acceleration that occurred during earthquake shaking. | USGS & CENC | 1000 m |
|  | PGV | Maximum ground velocity that occurred during earthquake shaking. |  |  |

Supplementary Table 5. VIFs of the PCIs after iterated multicollinearity and Pearson’s correlation analysis.

| PCI | Global  scale | Temperate  AH region | Cold  AH region | Equatorial  CP region | Temperate  CP region | Cold  CP region |
| --- | --- | --- | --- | --- | --- | --- |
| Slope | 7.81 | 8.13 | / | 6.40 | 4.70 | 3.51 |
| Aspect | 4.74 | 4.90 | 4.98 | 4.58 | 4.81 | 4.68 |
| Relief | / | / | 9.78 | 8.69 | / | / |
| TPI | 1.27 | 1.62 | 1.05 | 1.24 | 1.63 | 1.16 |
| Terrain roughness | 5.92 | 6.02 | 6.00 | 4.53 | 4.15 | / |
| Plan curvature | 1.00 | 1.00 | 1.04 | 1.00 | 1.00 | 1.00 |
| Profile curvature | / | / | / | / | / | / |
| Lithology | 3.37 | 2.76 | 2.96 | 3.60 | 6.87 | / |
| Soil type | 3.16 | 6.62 | 4.53 | 2.84 | 2.83 | 4.45 |
| Land cover | 3.52 | 3.55 | 3.76 | 4.64 | 3.76 | 2.51 |
| NDVI | 7.29 | 9.23 | 8.03 | 9.16 | 6.79 |  |
| TWI | 2.13 | 2.00 | 2.24 | 2.30 | 2.36 | 2.18 |
| Distance to river | 2.84 | 2.57 | 3.00 | 2.85 | 3.04 | 3.77 |
| Distance to fault | 1.96 | 2.48 | 2.15 | 2.68 | 2.10 | 2.84 |
| Fault mechanism | / | / | / | / | / | / |
| PGA | 4.11 | 3.30 | 5.80 | 4.86 | 3.84 | / |
| PGV | / | / | / | / | / | / |

**Reference**

1 Tadono T, Ishida H, Oda F *et al.* Precise global DEM generation by ALOS PRISM. *ISPRS Annals of the Photogrammetry, Remote Sensing and Spatial Information Sciences* 2014; **2**: 71-76.

2 Schwanghart W, Scherler D. TopoToolbox 2–MATLAB-based software for topographic analysis and modeling in Earth surface sciences. *Earth Surf Dyn* 2014; **2**: 1-7.

3 Hartmann J, Moosdorf N. The new global lithological map database GLiM: A representation of rock properties at the Earth surface. *Geochem Geophys Geosyst* 2012; **13**.

4 Sanchez P A, Ahamed S, Carré F *et al.* Digital soil map of the world. *Science* 2009; **325**: 680-681.

5 Zhang X, Zhao T, Xu H *et al.* GLC_FCS30D: the first global 30 m land-cover dynamics monitoring product with a fine classification system for the period from 1985 to 2022 generated using dense-time-series Landsat imagery and the continuous change-detection method. *Earth Syst Sci Data* 2024; **16**: 1353-1381.

6 Wu X, Xu X, Yu G *et al.* The China Active Faults Database (CAFD) and its web system. *Earth Syst Sci Data* 2024; **16**: 3391-3417.

7 Styron R, Pagani M. The GEM global active faults database. *Earthq Spectra* 2020; **36**: 160-180.

8 Meunier P, Hovius N, Haines J A. Topographic site effects and the location of earthquake induced landslides. *Earth Planet Sci Lett* 2008; **275**: 221-232.

9 Alberti S, Leshchinsky B, Roering J *et al.* Inversions of landslide strength as a proxy for subsurface weathering. *Nat Commun* 2022; **13**: 6049.

10 Pardoux É, Veretennikov Y. On the Poisson equation and diffusion approximation. I. *The Annals of Probability* 2001; **29**: 1061-1085.

11 Hendrycks D, Gimpel K. Gaussian error linear units (gelus). arXiv: 160608415.

12 Woo S, Park J, Lee J-Y, Kweon I S. Cbam: Convolutional block attention module. 2018: 3-19.

13 Lavé J, Avouac J P. Fluvial incision and tectonic uplift across the Himalayas of central Nepal. *J Geophys Res-Solid Earth* 2001; **106**: 26561-26591.

14 Graves R W, Pitarka A. Broadband ground-motion simulation using a hybrid approach. *Bull Seismol Soc Amer* 2010; **100**: 2095-2123.

15 Graves R, Pitarka A. Refinements to the Graves and Pitarka (2010) broadband ground‐motion simulation method. *Seismol Res Lett* 2015; **86**: 75-80.

16 Wells D L, Coppersmith K J. New empirical relationships among magnitude, rupture length, rupture width, rupture area, and surface displacement. *Bull Seismol Soc Amer* 1994; **84**: 974-1002.

17 Zhang W, Chen X. Traction image method for irregular free surface boundaries in finite difference seismic wave simulation. *Geophys J Int* 2006; **167**: 337-353.

18 Zhang W, Zhang Z, Chen X. Three-dimensional elastic wave numerical modelling in the presence of surface topography by a collocated-grid finite-difference method on curvilinear grids. *Geophys J Int* 2012; **190**: 358-378.

19 Liu Y, Yu Z, Zhang Z *et al.* The high-resolution community velocity model V2.0 of southwest China, constructed by joint body and surface wave tomography of data recorded at temporary dense arrays. *Sci China-Earth Sci* 2023; **66**: 2368-2385.

20 Motazedian D, Atkinson G M. Stochastic finite-fault modeling based on a dynamic corner frequency. *Bull Seismol Soc Amer* 2005; **95**: 995-1010.

21 Atkinson G M, Boore D M. Earthquake ground-motion prediction equations for eastern North America. *Bull Seismol Soc Amer* 2006; **96**: 2181-2205.

22 Science Data Catalog. *Landslides from the February 4, 1976, Guatemala earthquake.* https://data.usgs.gov/datacatalog/data/USGS:5874b3d4e4b0a829a320bb73 (30 April 2025, date last accessed).

23 Science Data Catalog. *Landslides triggered by the Coalinga, California, Earthquake of May 2, 1983.* https://data.usgs.gov/datacatalog/data/USGS:5874a164e4b0a829a320bb10 (30 April 2025, date last accessed).

24 Science Data Catalog. *Landslides generated by the Loma Prieta, California, Earthquake of October 17, 1989.* https://data.usgs.gov/datacatalog/data/USGS:5874bd02e4b0a829a320bbb4 (30 April 2025, date last accessed).

25 Marc O, Hovius N, Meunier P *et al.* A seismologically consistent expression for the total area and volume of earthquake‐triggered landsliding. *J Geophys Res-Earth Surf* 2016; **121**: 640-663.

26 Science Data Catalog. *Inventory of landslides triggered by the 1994 Northridge, California earthquake.* https://data.usgs.gov/datacatalog/data/USGS:58595917e4b03639a6025f30 (30 April 2025, date last accessed).

27 Science Data Catalog. *Landslides triggered by the January 16, 1995, M 6.9 Kobe, Japan Earthquake.* https://data.usgs.gov/datacatalog/data/USGS:58b538b7e4b01ccd54fddf59 (30 April 2025, date last accessed).

28 Marzorati S, Luzi L, De Amicis M. Rock falls induced by earthquakes: a statistical approach. *Soil Dyn Earthq Eng* 2002; **22**: 565-577.

29 Science Data Catalog. *Landslides Triggered by Jueili Earthquake.* https://data.usgs.gov/datacatalog/data/USGS:5b5a20eee4b0610d7f4dcc52 (30 April 2025, date last accessed).

30 Hung J J. Chi-Chi earthquake induced landslides in Taiwan. *Earthquake Engineering and Engineering Seismology* 2000; **2**: 25-33.

31 Gorum T, Korup O, van Westen C J *et al.* Why so few? Landslides triggered by the 2002 Denali earthquake, Alaska. *Quat Sci Rev* 2014; **95**: 80-94.

32 Papathanassiou G, Valkaniotis S, Ganas A *et al.* The November 17th, 2015 Lefkada (Greece) strike-slip earthquake: Field mapping of generated failures and assessment of macroseismic intensity ESI-07. *Eng Geol* 2017; **220**: 13-30.

33 Sekiguchi T, Sato H. Feature and distribution of landslides induced by the Mid Niigata Prefecture Earthquake in 2004, Japan. *Journal of the Japan Landslide Society* 2006; **43**: 142-154.

34 Basharat M, Ali A, Jadoon I A K *et al.* Using PCA in evaluating event-controlling attributes of landsliding in the 2005 Kashmir earthquake region, NW Himalayas, Pakistan. *Nat Hazards* 2016; **81**: 1999-2017.

35 Harp E L, Hartzell S H, Jibson R W *et al.* Ramirez-Guzman, R. G. Schmitt, Relation of landslides triggered by the Kiholo Bay earthquake to modeled ground motion. *Bull Seismol Soc Amer* 2014; **104**: 2529-2540.

36 Sepúlveda S A, Serey A, Lara M *et al.* Landslides induced by the April 2007 Aysén fjord earthquake, Chilean Patagonia. *Landslides* 2010; **7**: 483-492.

37 Xu C, Xu X, Yao X *et al.* Three (nearly) complete inventories of landslides triggered by the May 12, 2008 Wenchuan Mw 7.9 earthquake of China and their spatial distribution statistical analysis. *Landslides* 2014; **11**: 441-461.

38 Yagi H, Sato G, Higaki D *et al.* Distribution and characteristics of landslides induced by the Iwate-Miyagi Nairiku Earthquake in 2008 in Tohoku District, Northeast Japan. *Landslides* 2009; **6**: 335-344.

39 Martino S, Prestininzi A, Romeo R W. Earthquake-induced ground failures in Italy from a reviewed database. *Nat Hazards Earth Syst Sci* 2014; **14**: 799-814.

40 Xu C, Shyu J B H, Xu X. Landslides triggered by the 12 January 2010 Port-au-Prince, Haiti, Mw=7.0 earthquake: visual interpretation, inventory compiling, and spatial distribution statistical analysis. *Nat Hazards Earth Syst Sci* 2014; **14**: 1789-1818.

41 Wartman J, Dunham L, Tiwari B *et al.* Landslides in eastern Honshu induced by the 2011 Tohoku earthquake. *Bull Seismol Soc Amer* 2013; **103**: 1503-1521.

42 Alfaro P, Delgado J, García-Tortosa F J *et al.* Widespread landslides induced by the Mw 5.1 earthquake of 11 May 2011 in Lorca, SE Spain. *Eng Geol* 2012; **137**: 40-52.

43 Xu C, Xu X, Shyu J B H. Database and spatial distribution of landslides triggered by the Lushan, China Mw 6.6 earthquake of 20 April 2013. *Geomorphology* 2015; **248**: 77-92.

44 Xu C, Xu X, Shyu J B H *et al.* Landslides triggered by the 22 July 2013 Minxian-Zhangxian, China, Mw 5.9 earthquake: Inventory compiling and spatial distribution analysis. *J Asian Earth Sci* 2014; **92**: 125-142.

45 Xu C, Xu X, Shen L *et al.* Inventory of landslides triggered by the 2014 Ms 6.5 Ludian earthquake and its implications on several earthquake parameters. *Seismology and Geology* 2014; **36**: 1186-1203.

46 Valagussa A, Frattini P, Valbuzzi E *et al.* Role of landslides on the volume balance of the Nepal 2015 earthquake sequence. *Sci Rep* 2021; **11**: 3434.

47 Martino S. Eartquake-induced landslides in Italy. From the distribution of effects to the hazard mapping. *Ital J Eng Geol Env* 2017; **1**: 53-67.

48 Tanyaş H, Görüm T, Fadel I *et al.* An open dataset for landslides triggered by the 2016 Mw 7.8 Kaikōura earthquake, New Zealand. *Landslides* 2022; **19**: 1405-1420.

49 Fan X, Scaringi G, Xu Q *et al.* Coseismic landslides triggered by the 8th August 2017 Ms 7.0 Jiuzhaigou earthquake (Sichuan, China): factors controlling their spatial distribution and implications for the seismogenic blind fault identification. *Landslides* 2018; **15**: 967-983.

50 Hu K, Zhang X, You Y *et al.* Landslides and dammed lakes triggered by the 2017 Ms 6.9 Milin earthquake in the Tsangpo gorge. *Landslides* 2019; **16**: 993-1001.

51 Tanyaş H, Hill K, Mahoney L *et al.* The world's second-largest, recorded landslide event: Lessons learnt from the landslides triggered during and after the 2018 Mw 7.5 Papua New Guinea earthquake. *Eng Geol* 2022; **297**: 106504.

52 Ferrario M. Landslides triggered by multiple earthquakes: insights from the 2018 Lombok (Indonesia) events. *Nat Hazards* 2019; **98**: 575-592.

53 Wang F, Fan X, Yunus A P *et al.* Coseismic landslides triggered by the 2018 Hokkaido, Japan (Mw 6.6), earthquake: spatial distribution, controlling factors, and possible failure mechanism. *Landslides* 2019; **16**: 1551-1566.

54 Zhao B. Landslides triggered by the 2018 Mw 7.5 Palu supershear earthquake in Indonesia. *Eng Geol* 2021; **294**: 106406.

55 Havenith H B, Guerrier K, Schlögel R *et al.* Earthquake-induced landslides in Haiti: analysis of seismotectonic and possible climatic influences. *Nat Hazards Earth Syst Sci* 2022; **22**: 3361-3384.

56 Fan X, Fang C, Dai L *et al.* Near real time prediction of spatial distribution probability of earthquake-induced landslides-Take the Lushan Earthquake on June 1, 2022 as an example. *Journal of Engineering Geology* 2022; **30**: 729-739.

57 Dai L, Fan X, Wang X *et al.* Coseismic landslides triggered by the 2022 Luding Ms 6.8 earthquake, China. *Landslides* 2023; **20**: 1277-1292.
